# Extracellular vesicles containing MFGE8 from colorectal cancer facilitate macrophage efferocytosis

**DOI:** 10.1186/s12964-024-01669-9

**Published:** 2024-05-27

**Authors:** Zhixin Ma, Yu Sun, Yang Yu, Wenjun Xiao, Zhijie Xiao, Tianyu Zhong, Xi Xiang, Zhigang Li

**Affiliations:** 1 Scientific Research Center, The Seventh Affiliated Hospital, Sun Yat-sen University, Shenzhen, 518107 China; 2 Guangdong Provincial Key Laboratory of Digestive Cancer Research, Digestive Diseases Center, The Seventh Affiliated Hospital, Sun Yat-sen University, Shenzhen, 518107 China; 3 Department of Laboratory Medicine, First Affiliated Hospital of Gannan Medical University, Ganzhou, 341004 China; 4 Shenzhen Key Laboratory of Chinese Medicine Active Substance Screening and Translational Research, Shenzhen, 518107 China

**Keywords:** Colorectal cancer, Extracellular vesicle, MFGE8, Macrophage, Efferocytosis

## Abstract

**Background:**

Colorectal cancer (CRC) commonly exhibits tolerance to cisplatin treatment, but the underlying mechanisms remain unclear. Within the tumor microenvironment, macrophages play a role in resisting the cytotoxic effects of chemotherapy by engaging in efferocytosis to clear apoptotic cells induced by chemotherapeutic agents. The involvement of extracellular vesicles (EVs), an intercellular communicator within the tumor microenvironment, in regulating the efferocytosis for the promotion of drug resistance has not been thoroughly investigated.

**Methods:**

We constructed GFP fluorescent-expressing CRC cell lines (including GFP-CT26 and GFP-MC38) to detect macrophage efferocytosis through flow cytometric analysis. We isolated and purified CRC-secreted EVs using a multi-step ultracentrifugation method and identified them through electron microscopy and nanoflow cytometry. Proteomic analysis was conducted to identify the protein molecules carried by CRC-EVs. MFGE8 knockout CRC cell lines were constructed using CRISPR-Cas9, and their effects were validated through in vitro and in vivo experiments using Western blotting, immunofluorescence, and flow cytometric analysis, confirming that these EVs activate the macrophage αvβ3-Src-FAK-STAT3 signaling pathway, thereby promoting efferocytosis.

**Results:**

In this study, we found that CRC-derived EVs (CRC-EVs) enhanced macrophage efferocytosis of cisplatin-induced apoptotic CRC cells. Analysis of The Cancer Genome Atlas (TCGA) database revealed a high expression of the efferocytosis-associated gene MFGE8 in CRC patients, suggesting a poorer prognosis. Additionally, mass spectrometry-based proteomic analysis identified a high abundance of MFGE8 protein in CRC-EVs. Utilizing CRISPR-Cas9 gene edition system, we generated MFGE8-knockout CRC cells, demonstrating that their EVs fail to upregulate macrophage efferocytosis in vitro and in vivo. Furthermore, we demonstrated that MFGE8 in CRC-EVs stimulated macrophage efferocytosis by increasing the expression of αvβ3 on the cell surface, thereby activating the intracellular Src-FAK-STAT3 signaling pathway.

**Conclusions:**

Therefore, this study highlighted a mechanism in CRC-EVs carrying MFGE8 activated the macrophage efferocytosis. This activation promoted the clearance of cisplatin-induced apoptotic CRC cells, contributing to CRC resistance against cisplatin. These findings provide novel insights into the potential synergistic application of chemotherapy drugs, EVs inhibitors, and efferocytosis antagonists for CRC treatment.

**Supplementary Information:**

The online version contains supplementary material available at 10.1186/s12964-024-01669-9.

## Background

According to recent cancer statistics, colorectal cancer (CRC) is the third most prevalent and second most lethal cancer in the world [1]. Despite the advancements in surgical procedures and the widespread use of adjuvant chemotherapy, the 5-year survival rate of CRC patients stands at 65% on average [2]. However, the outlook remains grim for patients with distant metastases or inoperable tumors, who have a 5-year survival rate of just 15% [2]. Cisplatin is a chemotherapy drug used to treat various types of cancer, such as bladder, ovarian, and testicular cancers [3]. However, its therapeutic efficacy in CRC is not pronounced, as many CRC patients exhibit the resistance to cisplatin. Therefore, the combination of cisplatin with other drugs proves effective in the treatment of CRC. For example, the combined application of curcumin with cisplatin effectively inhibits CRC through MicroRNA-137-Glutaminase axis [4]; Arctigenin increased the sensitivity of CRC resistant to cisplatin through the activation of autophagy [5]; And metformin up-regulated the sensitivity of CRC to cisplatin by activating PI3K/Akt pathway [6]. However, it remains unknown how CRC regulates macrophage clearance of cisplatin-induced apoptotic cells within the tumor microenvironment, consequently promoting its progression.

Extracellular vesicles (EVs), which act as intercellular mediators between tumor and immune cells, are a type of nanoparticle with bilayer lipid membranes [7, 8]. These vesicles are classified into exosomes (50–150 nm), microvesicles (200–800 nm), and large EVs (≥ 1000 nm, such as apoptotic bodies) based on their diameter differences [9]. EVs contain a rich mixture of molecular components, including miRNAs, lncRNAs, proteins, and lipids [10]. Once EVs are taken up by target cells, these biologically active substances can trigger the activation of related signaling pathways [11]. Thus, EVs play a crucial role in intercellular communication. In the tumor microenvironment (TME), tumor-derived EVs (TDEVs) can induce changes in macrophages, such as M2 polarization, increased PD-L1 expression, and the release of inflammatory factors [12–14]. On the other hand, EVs derived from tumor-associated macrophages (TAMs) can enhance tumor cell metastasis, resistance to chemotherapy, and angiogenesis [15–17]. Overall, the biological effects of EVs between tumor cells and macrophages are mainly immunosuppressive and tumor-promoting.

It has been established that TAMs play an important role in the process of efferocytosis, which is the removal of apoptotic tumor cells. When chemotherapy, radiotherapy, or targeted therapy causes tumor cells to undergo apoptosis, a large number of chemokines are released, which attract macrophages to the site. Meanwhile, apoptotic tumor cells display “eat-me” signals such as phosphatidylserine and calreticulin, which are recognized by receptors in macrophages responsible for efferocytosis, such as milk fat globule-epidermal growth factor-factor 8 (MFGE8), MerTK, Axl, and Tyro3 [18–21]. The interaction between these ligands and receptors leads to the engulfment of the apoptotic cells. Efferocytosis helps to reduce the release of chemotactic factors, establish a tumor-tolerant immunosuppressive environment, and prevent secondary necrosis [22]. Previous studies have shown that efferocytosis is associated with resistance to chemotherapy and affects clinical outcomes in multiple cancers [23–26]. However, the impact of TDEVs on the regulation of macrophage efferocytosis remains unclear.

In this study, we demonstrated that EVs derived from colorectal cancer (CRC-EVs) have the ability to enhance the efferocytosis of apoptotic tumor cells by macrophages. Our findings showed that MFGE8, a component present in the CRC-EVs, was responsible for enhancing macrophage efferocytosis. MFGE8 is a glycoprotein known for its role in regulating immune tolerance, particularly through promoting the phagocytosis of apoptotic cells [27]. Its structure is comprised of a mucin-like domain, two repetitive discoidin-like structural domains (C domains), and two repetitive EGF-like structural domains. A specific integrin-binding motif in the EGF-like domain binds to MFGE8, enabling integrin-mediated signaling and enhancing cell adhesion of αvβ3 and αvβ5 integrin heterodimers [28]. MFGE8 binds phosphatidylserine present on apoptotic cells to the αvβ3 integrins on phagocytes, thus enhancing the clearance of these cells [29]. Besides efferocytosis, MFGE8 can also promote angiogenesis and intestinal mucosal repair [30, 31]. Previous studies indicated that high levels of MFGE8 have been linked to poor prognosis and overexpression of MFGE8 has been shown to drive CRC progression [32, 33]. Our findings revealed that MFGE8 in the CRC-EVs increased macrophage efferocytosis of cisplatin-induced apoptotic tumor cells through the αvβ3-Src-FAK-STAT3 signaling pathway. This study sheds light on a novel mechanism wherein CRC-EVs participate in macrophage efferocytosis, contributing to cisplatin-induced CRC apoptosis.

## Methods

### Cell lines and cell culture

CT26 mouse colorectal cancer cells were obtained from Procell Life & Technology Co., Ltd., while MC38 mouse colorectal cancer cells were sourced from the Chinese Academy of Sciences. The cells were cultured in a 5% CO2 incubator at 37 °C, in DMEM (Biochannel, BC-M-005) supplemented with 10% fetal bovine serum (Biochannel, BC-SE-FBS01) and 1% penicillin/streptomycin (Biosharp, BL505A).

### Bone marrow-derived macrophage culture

The isolation and cultivation of bone marrow-derived macrophages (BMDMs) followed a previously described method [34]. Briefly, C57BL/6 mice (8–12 weeks) were euthanized, and the thigh muscle was removed to access the bone marrow in the femur and tibia, which was then flushed with DMEM. After centrifugation, the supernatant was discarded and the cells were lysed using red blood cell lysis buffer. The remaining cells were re-suspended in DMEM containing 10% FBS, 1% penicillin/streptomycin, and 20 ng/ml M-CSF (Sino Biological, 51,112-MNAH) and seeded in 12-well plates at a concentration of 0.3 × 10^6^/ml (1 ml per well). After 3 days, a half volume of MCSF medium was added, and the BMDMs were fully differentiated and ready for use by day 7. The animal procedures were approved by the Institutional Animal Care and Use Committees of Sun Yat-sen University.

### Construction of apoptotic tumor cells

In the study, cisplatin (Med Chem Express, HY-17,394) was used to trigger apoptosis in CT26 and MC38 cells. After the tumor cells reached 60-70% confluence, they were exposed to 0–45 µM cisplatin for a period of 48 h. To confirm the presence of apoptosis, the tumor cells were then stained using the FITC-Annexin V/APC-PI Apoptosis Detection Kit (YEASEN, 40302ES60). Once the apoptosis rate reached more than 80%, the cells were deemed suitable for use in experiments. Therefore, a routine dose of 45 µM cisplatin was used for 48 h to induce apoptosis in the tumor cells.

To facilitate experimental observation, we constructed CT26 and MC38 cells stably expressing green fluorescent protein (GFP-CT26 and GFP-MC38). Briefly, the cells were infected with GFP-containing lentiviruses, and after 48 h, a single-cell clone was selected based on fluorescence microscopy and amplified in culture. The morphology and growth of the GFP cells were found to be identical to those of the wild-type cells, so the same amount of cisplatin was used to construct apoptotic GFP cells for further analysis.

### Extracellular vesicle isolation and characterization

The isolation of EVs from CT26 and MC38 cells was performed using multi-step ultracentrifugation [35]. The procedure involved growing the cells to 60–70% confluency, replacing the medium with serum-free DMEM for 24 h, and collecting the culture medium. The medium was then centrifuged at 3000 × g for 15 min at 4 °C to remove cellular debris, followed by another centrifugation at 10,000 × g for 30 min to remove larger microvesicles and apoptotic bodies. The supernatant was then subjected to ultra-centrifugation at 100,000 × g for 3 h at 4 °C to obtain the EV pellets. The EV pellets were resuspended in PBS and used for further analysis. The EV identity was confirmed through various methods including electron microscopy, western blot, and nano-flow cytometry. Electron microscopy was used to observe the morphology of the EVs, while western blot was used to detect EV marker proteins such as CD9, CD81, and TSG101. Nano-flow cytometry was used to measure the size and concentration of the EVs. The quantity of EVs was also measured using the BCA Protein Assay Kit (Solarbio, PC0020).

### Western blot

The cells underwent lysis using RIPA buffer (25 mM Tris-Cl, pH 7.4, 150 mM NaCl, 1 mM EDTA, 5% glycerol, 1% NP-40) supplemented with protease inhibitor cocktail and PMSF. Protein concentrations were determined using a BCA protein assay kit. Equal amounts of protein from each sample were loaded onto a 10% SDS-PAGE gel and separated. The proteins were then transferred from the gel to a PVDF membrane using electrotransfer in a transfer buffer (25 mM Tris, 192 mM glycine, 20% methanol). After blocking with 5% nonfat milk for 1 h at room temperature, the membrane was incubated overnight at 4 °C with primary antibodies targeting CD9 (Santa Cruz Biotechnology, sc-13,118), CD63 (Santa Cruz Biotechnology, sc-5275), TSG101 (Santa Cruz Biotechnology, sc-7964), Mitofilin (Proteintech, 10179-1-AP), MFGE8 (Santa Cruz Biotechnology, sc-271,574), p-Src (Cell Signaling Technology, 6943 S), t-Src (Cell Signaling Technology, 2109 S), p-FAK (Cell Signaling Technology, 3283 S), t-FAK (Cell Signaling Technology, 3285 S), p-STAT3 (Cell Signaling Technology, 9145), t-STAT3 (Cell Signaling Technology, 9139), and GAPDH (Abbkine, ABL1025). Following washing in TBST (TBS with 0.1% Tween-20), the membrane was incubated with appropriate HRP-conjugated secondary antibodies for 1 h. Protein bands were visualized using a ChemiDoc Imaging System from Bio-Rad and quantified using ImageJ version 1.50i. All uncropped Western Blot images can be found in the supplementary file “Uncropped Western Blot”.

### Mass spectrometry-based proteomic analysis

The CT26-EVs samples were extracted through the multi-step ultracentrifugation method. These samples were then processed for label-free mass spectrometry analysis using the EasyPep™ MS Sample Prep Kits (ThermoFisher Scientific, A40006). The steps involved in the preparation of the samples included dilution of the EV samples in Lysis Solution, determination of protein concentration using the BCA kit, addition of Reduction Solution and Alkylation Solution to the samples, incubation of the samples at 95 °C for 10 min to reduce and alkalize the protein, addition of reconstituted enzyme solution to digest the protein, and a “clean-up peptides” procedure. The resulting samples were sent for LC-MS/MS-based label-free quantitation detection (Agilent 6460 Triple Quadrupole LC/MS), and three biological replicates were performed. The proteins were identified by searching the human UniProt database using MaxQuant software, and the Label-Free Quantitation (LFQ) algorithm was used for quantitative analysis.

### Flow cytometry

Flow cytometry was performed to assess efferocytosis, as described in previous studies [36]. The procedure involved the pre-incubation of BMDMs with 10 µg/ml CT26-EVs or MC38-EVs for 24 h, while the control group was treated with PBS. Then, both the control and EVs-treated BMDMs were incubated with apoptotic tumor cells in a 1:3 ratio for 4 h. After being washed with PBS, the cells were collected and stained with APC-F4/80 antibody (Multi Sciences, AM048005) for 30 min to label the BMDMs. The APC and GFP double-positive BMDMs were then analyzed using flow cytometry. The percentage of GFP-positive cells within the APC-positive population was calculated using FlowJo v10.6.2 software.

### Confocal immunofluorescence

The BMDMs were cultured in 12-well glass-bottomed dishes for 7 days. Prior to the efferocytosis assay, the BMDMs were pre-incubated for 24 h with 10 µg/ml of either CT26-EVs or MC38-EVs, while the control group was given PBS. Afterwards, the control and EVs-treated BMDMs were incubated with apoptotic tumor cells in a 1:3 ratio for 4 h. The cells were then washed three times with PBS and fixed with 4% paraformaldehyde for 15 min at room temperature. The cells were permeabilized with 0.3% Triton X-100 in PBS for 15 min, followed by blocking with 3% BSA in PBS for 0.5 h at room temperature. The BMDMs were then incubated with F4/80 antibody (Santa Cruz Biotechnology, sc-377,009) overnight at 4 °C and later with APC-conjugated secondary antibody (HuaAn Biotechnology, HA1127) for 2 h at room temperature. The cells were finally mounted with a mounting medium containing DAPI (Solarbio, S2110) and imaged using confocal fluorescence microscopy. The efferocytosis rate of apoptotic tumor cells in the BMDMs was quantified using Zen Pro and ImageJ software.

To confirm the internalization of the CRC-EVs into the BMDMs, the CT26-EVs and MC38-EVs were labeled with PKH67 Green Fluorescent Cell Linker Mini Kit (fluorescence, PKH67). The EVs were stained for 20 min as per the instructions and then added to the BMDMs for 30 min. The cells were permeabilized with 0.3% Triton X-100 in PBS for 15 min, followed by blocking with 3% BSA in PBS for 0.5 h. The BMDMs were incubated with the F4/80 antibody overnight at 4 °C, followed by incubation with APC-conjugated secondary antibody for 2 h at room temperature. Finally, the cells were mounted with a mounting medium containing DAPI and imaged using confocal fluorescence microscopy.

### RNA extraction and quantitative real-time PCR

To extract RNA, the treated cells were lysed with Trlquick reagent (R1100, Solarbio), and total RNA was obtained through a series of centrifugations according to the specified protocol. The concentration of the extracted RNA was determined by measuring the spectrophotometric absorption at 260/280 nm. The RNA was then reverse-transcribed into cDNA using the 5 × Evo M-MLV RT Master Mix (AG11706, Accurate Biology), with reaction conditions of 37°C for 15 minutes and 85°C for 5 seconds. The resulting cDNA was diluted in water, and 10 ng was used for each quantitative RT-PCR (qRT-PCR) analysis. The qRT-PCR was carried out using a 2x Taq Pro Universal SYBR qPCR Master Mix (Q712-02, Vazyme) under the following conditions: 95°C for 30 seconds, followed by 95°C for 10 seconds and 60°C for 30 seconds, repeated for 40 cycles. The primers used for gene amplification were: MFGE8, forward 5’- AGATGCGGGTATCAGGTGTGA-3’ and reverse 5’- GGGGCTCAGAACATCCGTG-3’; and GAPDH, forward 5’- CATCACTGCCACCCAGAAGACTG-3’ and reverse 5’- ATGCCAGTGAGCTTCCCGTTCAG-3’. The fold change was calculated using the ΔΔ threshold cycle method, normalized to the control group.

### CRISPR-Cas9 RNP nucleofection for Mfge8 knockout

In this study, we employed CRISPR-Cas9 ribonucleoprotein (RNP) nucleofection to knock out Mfge8 in CT26 and MC38 cells, following a previously established protocol [37]. To achieve this, we obtained two guide RNAs (gRNAs) from the GenScript Company (Nanjing, China), which were designed to target the mouse Mfge8 gene. The sequences for the gRNAs were Mfge8-gRNA1: TGGCTCGTCTGTACCGCACAGG, and Mfge8-gRNA2: TACCCTGTTTCGTGCCACCGCGG. Prior to nucleofection, 0.5 µl of gRNA1 and 0.5 µl of gRNA2 (3.2 µg/µl) were mixed with 0.5 µl of spCas9 protein (from Integrated DNA Technologies, Iowa, USA) and allowed to form an RNP complex at room temperature for 10 min (up to 1 h). A control group was included, in which the gRNA mixture was nucleofected without the spCas9 protein.

We utilized the P3 Primary Cell 4D-Nucleofector™ X Kit (Lonza, Switzerland) to perform nucleofection as per the manufacturer’s instructions. The cells were trypsinized, washed with PBS twice, and 200,000 cells were collected and suspended in 20 µl of Nucleofector™ Solution. The cell suspension was then mixed with the RNP complex. The nucleofection was carried out using the Lonza 4D-nucleofector, and the cells were transferred to a 12-well plate containing pre-warmed culture medium. All cells were harvested 48 hours after nucleofection for further analysis, which included DNA genotyping and qPCR. The primers used for Mfge8 genotyping after the knockout were Mfge8-scr-F: 5’-GATGGTCACTGTCTCCCTGC-3’ and Mfge8-scr-R: 5’-CATGGCAGACAGCACATAACTG-3’.

### In vivo study of peritoneal macrophage efferocytosis

In this study, we measured the percentage of tumor cells phagocytosed by peritoneal macrophages in mice, following intraperitoneal injection of apoptotic CT26 or MC38 cells. The method used was previously described [38]. Mice of 12 weeks old were given intraperitoneal injections of CT26-EVs or MC38-EVs at a dose of 50 µg per mouse in 200 µl of PBS for a period of 24 h. The control group received injections of PBS without EVs. Later, 2 × 10^6^ apoptotic CT26 or MC38 cells in 1 ml PBS were intraperitoneally injected into the mice. After 4 h, the mice were humanely euthanized, and a peritoneal lavage was performed using 50 ml of sterile PBS. The isolated cells were then stained with an APC-F4/80 antibody. The final step involved analysis of the percentage of peritoneal macrophage phagocytosis of the apoptotic tumor cells by flow cytometry. All animal procedures were approved by the Institutional Animal Care and Use Committees of Sun Yat-sen University.

### Statistical analysis

The statistical analysis was conducted using GraphPad Prism v.8.0. The results were presented as the mean ± standard error of mean (SEM) from a specified number of experiments. Statistical comparison was carried out using a one-way analysis of variance (ANOVA) with Tukey post-hoc test or an unpaired two-tailed Student’s t-test, and a *P*-value less than 0.05 was considered statistically significant.

## Results

### Macrophage efferocytosis of cisplatin-induced apoptotic CRC cells

**Fig. 1:**
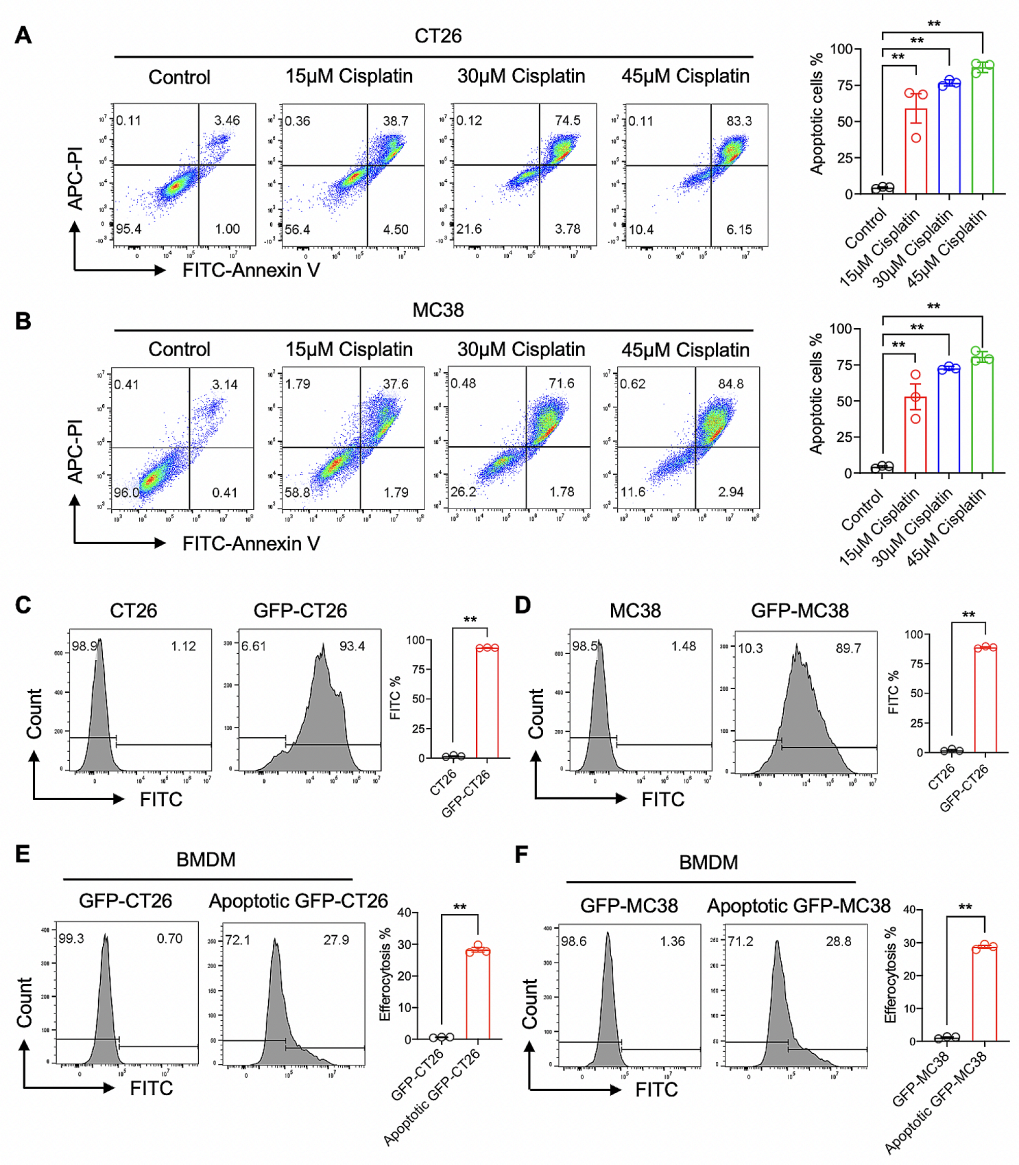
Macrophage efferocytosis of cisplatin-induced apoptotic CRC cells. (**A** and **B**) The apoptosis rates of CT26 and MC38 cells after exposure to cisplatin at concentrations of 0, 15, 30, or 45 µM for 48 h were determined by Annexin V and PI double staining and flow cytometry analysis. (**C** and **D**) Stable expression of GFP in CT26 and MC38 cells was established and verified by flow cytometry analysis of the percentage of the GFP + population. (**E**) The efferocytosis rate was evaluated by staining BMDMs with APC-F4/80 antibody after treatment with cisplatin-induced apoptotic GFP-CT26 cells (1:3 ratio for 4 h). The rate was determined by analyzing the percentage of the GFP + population in the APC-F4/80 + population via flow cytometry. (**F**) The efferocytosis rate was calculated by analyzing the percentage of the GFP + population in the APC-F4/80 + population after treatment with cisplatin-induced apoptotic GFP-MC38 cells (1:3 ratio for 4 h). Data was shown in mean ± SEM of *n* = 3 independent experiments per condition. **P < 0.01. Student’s t-test

We exposed the colorectal cancer (CRC) cells including CT26 and MC38 cells to cisplatin doses ranging from 0 to 45 µM for 48 h, and as seen in Fig. 1A and B, cisplatin induced apoptosis in the CT26 and MC38 cells in a dose-dependent manner. When treated with 45 µM cisplatin, the apoptosis rate was approximately 83.3% and 84.8% for the CT26 and MC38 cells, respectively. To further investigate the efferocytotic rate, we established CT26 and MC38 cell lines with stable GFP expression. By selecting GFP-positive monoclonal cells for expansion culture, over 90% of GFP-CT26 and GFP-MC38 cells displayed green fluorescence as seen in Figures S1A and S1B. Flow cytometry showed that the green fluorescence percentage of GFP-CT26 and GFP-MC38 cells was about 90% (Fig. 1C and D). We found that the growth status of GFP cells was identical to that of wild-type cells, so we treated the GFP cells with 45 µM cisplatin for 48 h to construct apoptotic tumor cells for later experiments.

Previous studies have demonstrated that macrophages can engulf apoptotic tumor cells during co-culture [36]. In this study, we added apoptotic CT26 or MC38 cells to BMDMs for 4 h and then collected the cells for F4/80 staining and flow cytometry analysis. The gating strategy for cell population analysis is shown in Supplementary Figure S2A, in which the F4/80 positive population is represented as macrophages. As seen in Fig. 1E and F, BMDMs efferocytosis of approximately 28% of apoptotic GFP-CT26 or GFP-MC38 cells after 4 h of co-culture. This model effectively showed that macrophages have the capability to perform efferocytosis on cisplatin-induced apoptotic CRC cells.

### CRC-EVs promoted macrophage efferocytosis

**Fig. 2:**
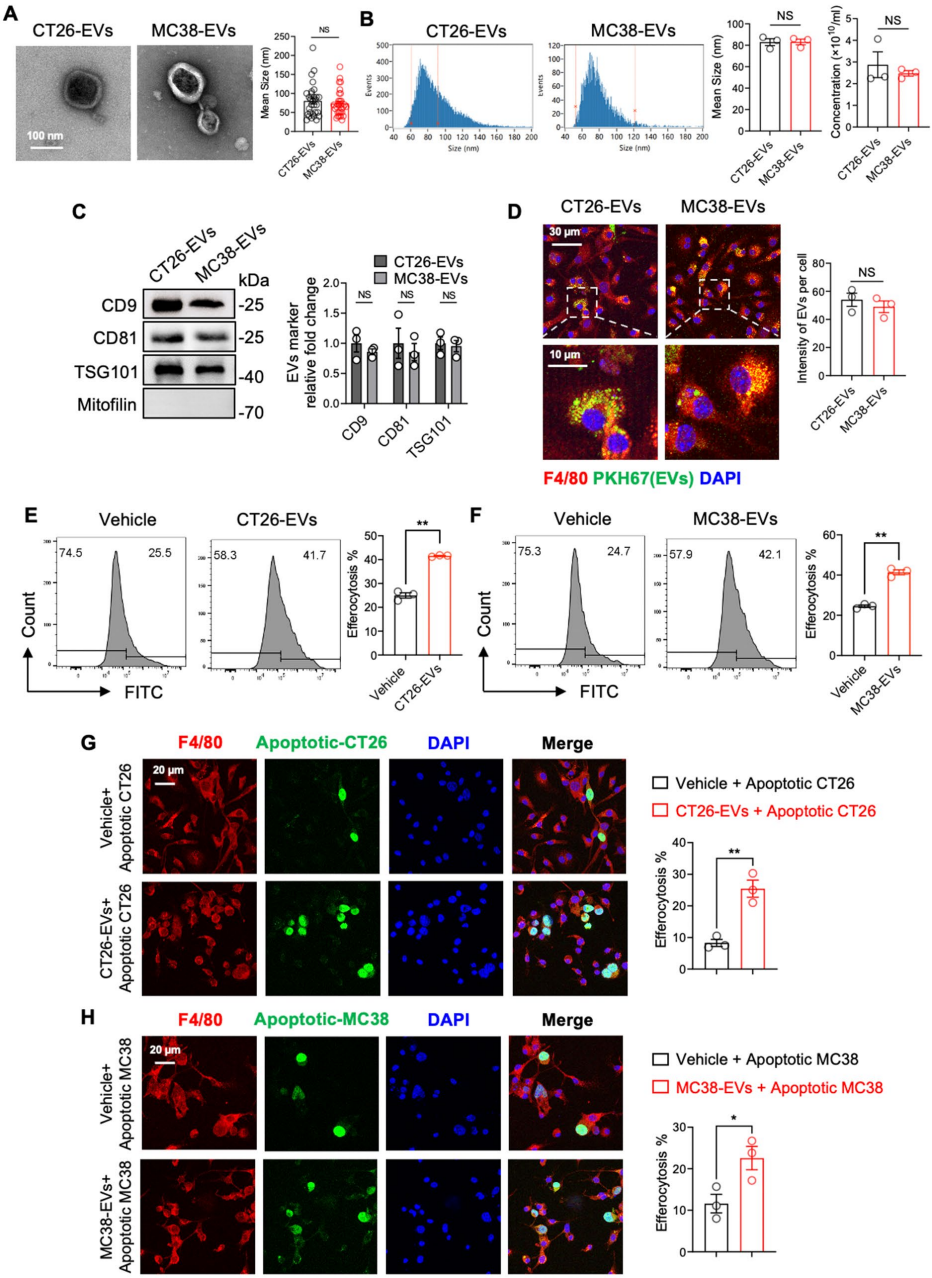
CRC-EVs promoted macrophage efferocytosis. (**A**) Electron microscopy images represented the morphology of EVs derived from CT26 and MC38 cells (CT26-EVs and MC38-EVs), and the sizes of CT26-EVs and MC38-EVs were determined using electron microscopy. (**B**) The size and concentration of CT26-EVs and MC38-EVs were analyzed using nano-flow cytometry. (**C**) Small EV markers (CD9, CD81, and TSG101) and the large EV marker (Mitofilin) were identified through western blot analysis in CT26-EVs and MC38-EVs. (**D**) Immunofluorescence images demonstrating the uptake of PKH67-labeled CRC-EVs by BMDMs were shown. The statistics represent the mean grayscale values of EVs within cells. (**E** to **H**) BMDMs were exposed to Vehicle (PBS), CT26-EVs (10 µg/ml), or MC38-EVs (10 µg/ml) for 24 h. Then, BMDMs were treated with cisplatin-induced apoptotic CT26 or MC38 cells in a 1:3 ratio for 4 h. BMDMs were stained with APC-F4/80 antibody and the efferocytosis rate was measured by determining the percentage of BMDMs that had efferocytosis of apoptotic tumor cells using flow cytometry or confocal fluorescence microscopy. (**E**) The percentage of BMDMs that had efferocytosis of apoptotic CT26 cells after exposure to Vehicle or CT26-EVs was analyzed using flow cytometry. (**F**) The efferocytosis rate of BMDMs after exposure to Vehicle or MC38-EVs was determined by flow cytometry. (**G**) Confocal microscopy was used to analyze the percentage of BMDMs that had efferocytosis of cisplatin-induced apoptotic CT26 cells after exposure to Vehicle or CT26-EVs. (**H**) The efferocytosis rate of BMDMs after exposure to Vehicle or MC38-EVs was analyzed using confocal microscopy. The efferocytosis percentage, as measured by the confocal fluorescence microscope described above, refers to the ratio of macrophages engulfing green fluorescence to all red-stained macrophages. All statistical graph was generated based on immunofluorescence images obtained from three independent replicate experiments, with an average calculated from three random fields for each experimental group. Data was shown in mean ± SEM of *n* = 3–10 independent experiments per condition. *P < 0.05 or **P < 0.01. Student’s t-test

CRC cells including CT26 and MC38 -derived extracellular vesicles (represented as CT26-EVs and MC38-EVs) were isolated from the culture medium using multi-step ultracentrifugation. Electron microscopy images showed that the CT26 and MC38 EVs had the typical “cup-sharp” shape (Fig. 2A), and their diameters were 80.33 ± 7.87 nm and 73.67 ± 6.22 nm respectively (Fig. 2A). Additionally, nano-flow cytometry showed the diameters of CT26 and MC38 EVs to be 82.94 ± 3.21 nm and 83.08 ± 2.64 nm, with concentrations of 2.87 ± 0.59 × 10^10^/ml and 2.48 ± 0.12 × 10^10^/ml respectively (Fig. 2B). Western blot analysis demonstrated that CT26 and MC38 EVs were enriched with CD9, CD81, and TSG101 (small EV positive markers), but lacked Mitofilin (a small EV negative marker) (Fig. 2C). These results suggest that the CT26 and MC38 EVs isolated using multi-step ultracentrifugation were mainly small EVs, also known as exosomes.

To observe the interaction between EVs and macrophages, we labeled the EVs with PKH67 and the macrophages with F4/80. Confocal fluorescence microscopy showed that the EVs were absorbed into the macrophages’ cytoplasm, indicating internalization (Fig. 2D). To assess the effect of CRC-EVs on macrophage efferocytosis, BMDMs were treated with 10 µg/ml of CT26-EVs for 24 h, and then co-cultured with apoptotic CT26 cells at a ratio of 1:3 for 4 h. As shown in Fig. 2E, the efferocytosis of apoptotic CT26 cells was significantly increased in the CT26-EVs treatment compared to the vehicle group. Similarly, MC38-EVs also increased the ability of BMDMs to efferocytosis of apoptotic MC38 cells (Fig. 2F). Confocal fluorescence microscopy revealed a significant increase in the number of apoptotic cells undergoing efferocytosis in the treatment groups (CT26-EVs and MC38-EVs), compared to the vehicle group where only a few apoptotic cells were observed to be undergoing efferocytosis (Fig. 2G and H). Calculating the percentage of macrophages involved in efferocytosis confirmed that both CT26-EVs and MC38-EVs significantly enhanced the macrophage efferocytosis ability (Fig. 2G and H). These results demonstrated that CRC-EVs can effectively enhance the macrophage efferocytosis of cisplatin-induced apoptotic CRC cells.

### CRC-EVs carried MFGE8 to promote macrophage efferocytosis

**Fig. 3:**
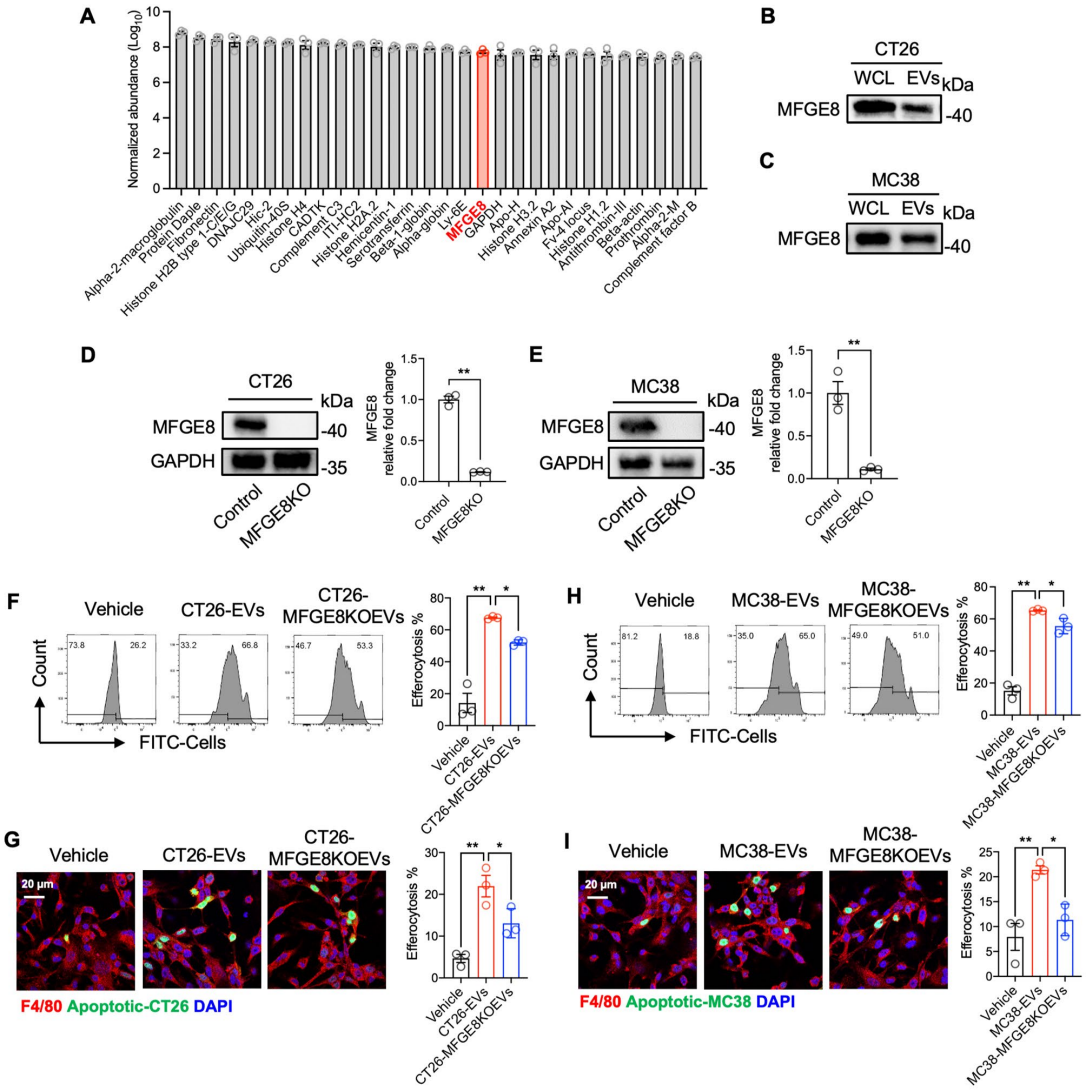
CRC-EVs carried MFGE8 to promote macrophage efferocytosis. (**A**) The histogram demonstrating the top 30 proteins with the highest concentration in the CT26-EVs proteomic analysis. (**B** and **C**) The MFGE8 expression in the whole cell lysates and the released EVs from CT26 and MC38 cells was measured by western blotting. (**D** and **E**) The protein expression of MFGE8 in CT26 and MC38 cells with MFGE8 knockouts was analyzed using western blotting. (**F** and **G**) BMDMs were treated with Vehicle (PBS), CT26-EVs (10 µg/ml) or MFGE8 knockout CT26-EVs (10 µg/ml) for 24 h, and then with apoptotic CT26 cells in a 1:3 ratio for 4 h. The percent of BMDMs that efferocytosis of the apoptotic CT26 cells was assessed by flow cytometry (**F**) and confocal fluorescence microscopy (**G**). (**H** and **I**) BMDMs were treated with PBS (control), MC38-EVs, or MFGE8 knockout MC38-EVs for 24 h and then with apoptotic MC38 cells for 4 h. The percent of BMDMs that efferocytosis of the apoptotic MC38 cells was measured by flow cytometry (**H**) and confocal fluorescence microscopy (**I**). The efferocytosis percentage, determined using the confocal fluorescence microscope described above, represents the proportion of macrophages engulfing green fluorescence to all red-stained macrophages. All statistical graphs were generated from immunofluorescence images obtained from three independent replicate experiments, with an average calculated from three random fields for each experimental group. Data was shown in mean ± SEM of *n* = 3–6 independent experiments per condition. *P < 0.05 or **P < 0.01. Student’s t-test or one-way ANOVA with Tukey post-hoc comparisons

To determine the presence of cargo proteins in CRC-EVs that promoted macrophage efferocytosis, we conducted mass spectrometry-based proteomic analysis of proteins isolated from CT26-EVs. The full list of proteins discovered in CT26-EVs can be found in the supplementary material “CT26-EVs MS Proteomic Data”. The top 30 most abundant proteins were displayed in Fig. 3A, and among them, we found that MFGE8 was the only molecule linked to efferocytosis. To verify the expression of MFGE8 in CRC-EVs, we performed a western blot analysis and found that MFGE8 was expressed not only in whole cell lysis but also in CT26-EVs and MC38-EVs (Fig. 3B and C). To further study the role of MFGE8 in CRC-EVs, we constructed MFGE8-knockout CT26 and MC38 cells (represented as CT26-MFGE8KO and MC38-MFGE8KO) using the CRISPR-Cas9 gene editing system. We then amplified the culture of monoclonal cells, which was confirmed by PCR (Fig. S3A). The mRNA and protein expression of MFGE8 were then determined using western blot and RT-qPCR, and as expected, MFGE8 was not detected in CT26-MFGE8KO and MC38-MFGE8KO cells (Fig. 3D and E, and S3B).

The EVs isolated from CT26-MFGE8KO and MC38-MFGE8KO cells were identified by nano-flow cytometry and western blot. The diameters of CT26-MFGE8KOEVs and MC38-MFGE8KOEVs were around 80 nm, and the concentrations of CT26-EVs and MC38-EVs were 3.03 ± 0.50 × 10^10^/ml and 3.67 ± 0.74 × 10^10^/ml, respectively (Fig. S3C). Both CT26-EVs and MC38-EVs were found to be enriched with CD9, CD81, and TSG101 (small EV positive markers), but not Mitofilin (a small EV negative marker), indicating that the MFGE8 knockout did not affect EV release in CT26 and MC38 cells.

To examine the impact of MFGE8-deficient EVs on macrophage efferocytosis, we exposed BMDMs to the same concentration (10 µg/ml) of CT26-EVs or CT26-MFGE8KOEVs for 24 h, followed by the addition of apoptotic CT26 cells for 4 h. The efferocytosis rate of apoptotic CT26 cells was significantly reduced in the CT26-MFGE8KOEVs treatment group compared to the CT26-EVs treatment group (Fig. 3F). The efferocytosis of apoptotic cells in the CT26-MFGE8KOEVs treatment group was also significantly lower than in the CT26-EVs treatment group, as demonstrated by immunofluorescence images (Fig. 3G). The same results were found for the efferocytosis of apoptotic MC38 cells, where MFGE8 knockout diminished MC38-EVs-mediated macrophage efferocytosis (Fig. 3H and I).

### High expression of MFGE8 in CRC is associated with poor prognosis

**Fig. 4:**
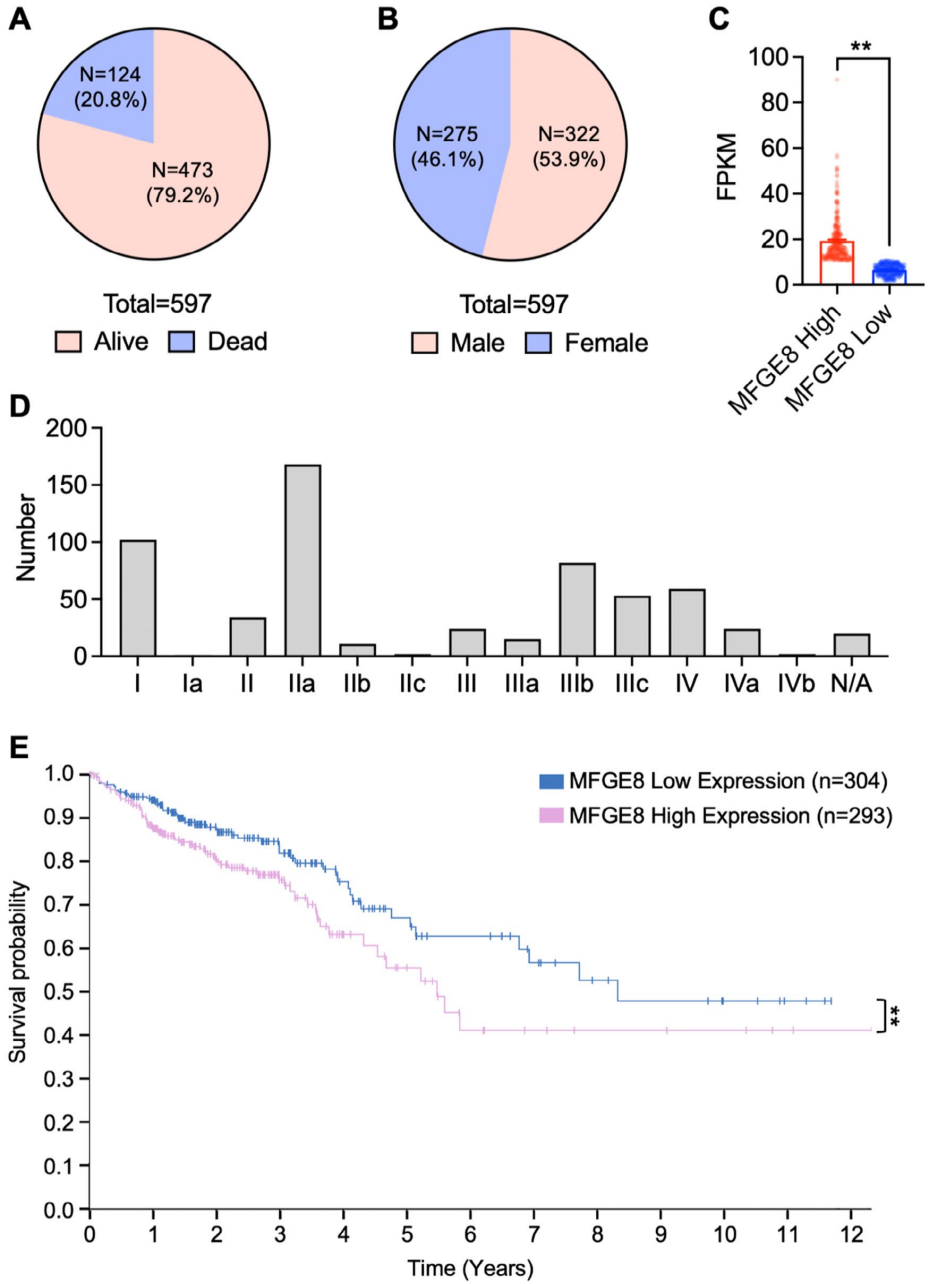
High expression of MFGE8 in CRC is associated with poor prognosis. (**A-B**) The alive-to-death ratio (**A**) and gender distribution (**B**) among a total of 597 CRC patients from the TCGA (The Cancer Genome Atlas) database. (**C**) CRC patients with MFGE8 FPKM values greater than 10.75 were categorized as high MFGE8 expression, while those with FPKM values less than 10.75 were categorized as low MFGE8 expression. (**D**) Distribution of patients across different clinical stages, including stages I-IV and N/A (not staged). (**E**) Survival regression curves were represented for CRC patients with high and low MFGE8 expression

The above data demonstrated that MFGE8 carried in CRC-EVs can participate in promoting macrophage efferocytosis of cisplatin-induced apoptotic CRC cells. Given that enhanced efferocytosis by macrophages is known to promote the progression of CRC, we sought to investigate whether the expression of MFGE8 in CRC is correlated with the clinical progression of CRC patients. Following an analysis of data from The Cancer Genome Atlas (TCGA) database (see Supplementary Materials - CRC patients MFGE8), we identified a total of 597 CRC patients, of which 473 (79.2%) had deceased, and 124 (20.8%) were alive (Fig. 4A). Among them, 275 were male (46.1%), and 322 were female (53.9%) (Fig. 4B). The best expression cutoff for FPKM (Fragments Per Kilobase of transcript per Million mapped reads) was determined to be 10.75, classifying patients with MFGE8 FPKM greater than 10.75 as high-expression CRC patients and those with MFGE8 FPKM less than 10.75 as low-expression CRC patients (Fig. 4C). The distribution of patients across different stages was represented in Fig. 4D, with the majority being in stage IIa. Survival analysis based on the relationship between MFGE8 expression and survival rates revealed that CRC patients with high expression of MFGE8 exhibited significantly lower survival rates, indicating a pronounced association with poor prognosis (Fig. 4E).

### MFGE8 in CRC-EVs upregulated macrophage αvβ3 expression

**Fig. 5:**
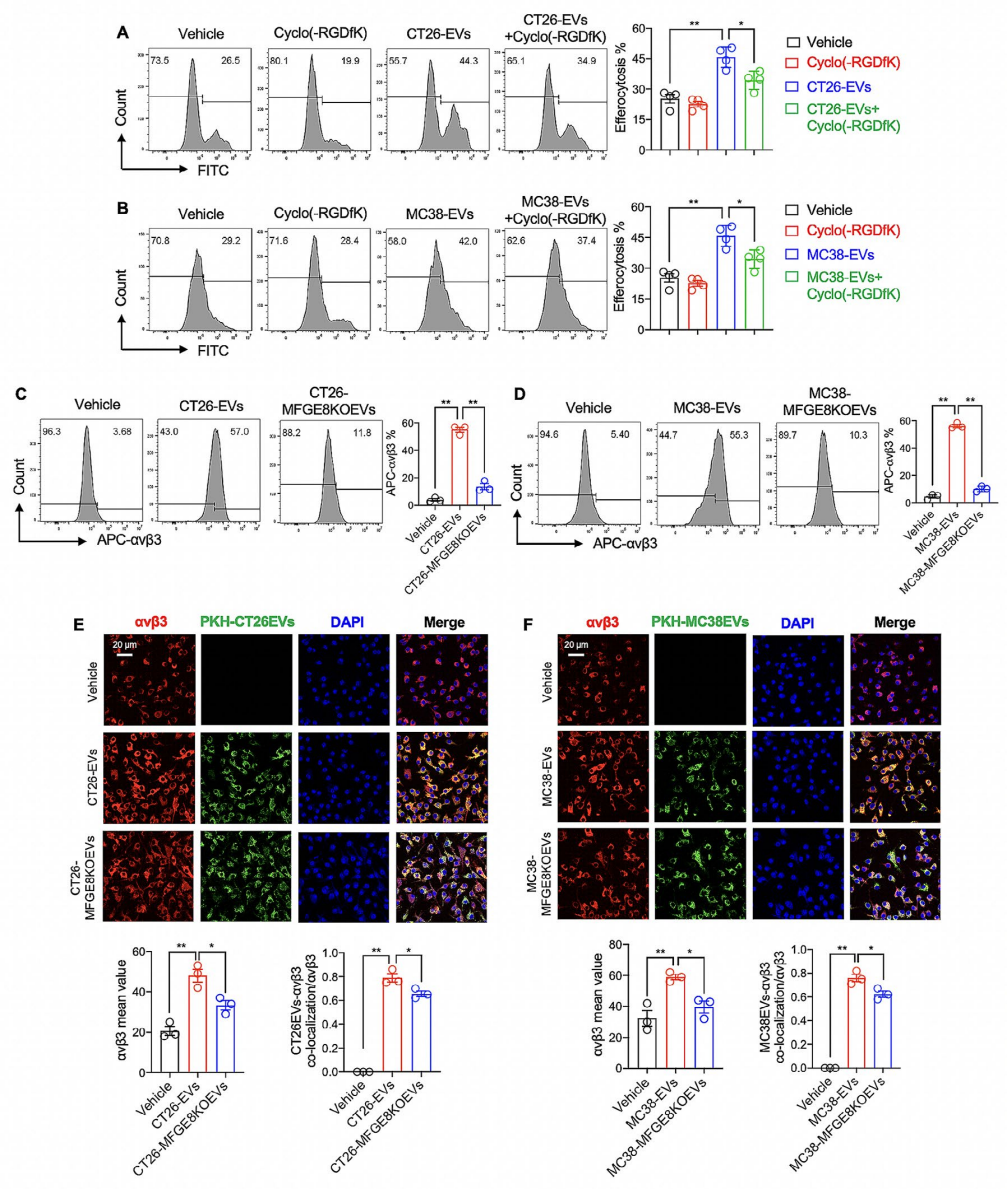
MFGE8 in CRC-EVs upregulated macrophage αvβ3 expression. (**A** and **B**) BMDMs were treated with Vehicle (DMSO), 20 µM Cyclo(-RGDfK), 10 µg/ml CRC-EVs (CT26-EVs or MC38-EVs), or 10 µg/ml CRC-EVs combined with 20 µM Cyclo(-RGDfK) for 24 h. Then, the BMDMs were exposed to cisplatin-induced apoptotic tumor cells in a 1:3 ratio for 4 h, and the efferocytosis percentage of the apoptotic tumor cells by the BMDMs was analyzed using flow cytometry. (**C** and **D**) BMDMs were treated with either Vehicle (PBS), 10 µg/ml CRC-EVs (CT26-EVs or MC38-EVs), or 10 µg/ml CRC-MFGE8KOEVs (CT26-MFGE8KOEVs or MC38-MFGE8KOEVs) for 24 h. The cell surface expression of αvβ3 on BMDMs was subsequently assessed using flow cytometry. (**E** and **F**) BMDMs were exposed to Vehicle (PBS), 10 µg/ml PKH67-labeled CRC-EVs (CT26-EVs or MC38-EVs), or 10 µg/ml PKH67-labeled CRC-MFGE8KOEVs (CT26-MFGE8KOEVs or MC38-MFGE8KOEVs) for 24 h. Following treatment, αvβ3 on BMDMs was stained with an αvβ3 antibody, and the co-localization of PKH67-labeled EVs and αvβ3 was measured using confocal microscopy. The intensity of αvβ3 fluorescence expression is assessed by computing the mean value of each image, while the co-localization ratio of PKH67-labeled EVs and αvβ3 is determined by comparing the overlapping fluorescence areas of PKH67-labeled EVs and αvβ3 to the total fluorescence area of αvβ3. All statistical graphs were derived from immunofluorescence images acquired from three independent replicate experiments, with averages computed from three random fields for each experimental group. Data was shown in mean ± SEM of *n* = 3–4 independent experiments per condition. *P < 0.05 or **P < 0.01. One-way ANOVA with Tukey post-hoc comparisons

The protein MFGE8 has been shown to bind to the integrin αvβ3, activating the STAT3 signaling pathway and leading to increased macrophage efferocytosis [39]. To determine if CRC-EVs modulated macrophage efferocytosis through the αvβ3, we pre-treated BMDMs with Cyclo(-RGDfK), a specific inhibitor of integrin αvβ3. The results showed that only Cyclo(-RGDfK) did not impact efferocytosis of apoptotic tumor cells by macrophages, but when combined with CT26-EVs or MC38-EVs, it reversed the efferocytosis effect induced by the CRC-EVs (including CT26-EVs and MC38-EVs) (Fig. 5A and B).

We then determined whether CRC-EVs could affect the expression of αvβ3 in macrophages. We found that CRC-EVs (including CT26-EVs and MC38-EVs) induced macrophages cell surface αvβ3 expression up-regulation (Fig. 5C and D). When knock-out of MFGE8 in CRC-EVs (including CT26-MFGE8KOEVs and MC38-MFGE8KOEVs), the expression of αvβ3 significantly down-regulation compared to CRC-EVs (Fig. 5C and D). We further utilized PKH67 labeling to trace CRC-EVs and investigated their co-localization with αvβ3. As shown in Fig. 5E and F, we found that CRC-EVs (CT26-EVs and MC38-EVs) induced an increase in the expression of αvβ3 and promoted co-localization of CRC-EVs with αvβ3 in macrophages. Furthermore, knock-out of MFGE8 in CRC-EVs (including CT26-MFGE8KOEVs and MC38-MFGE8KOEVs) led to a significant decrease in αvβ3 expression and reduced co-localization of CRC-EVs with αvβ3 in macrophages compared to wild-type CRC-EVs (Fig. 5E and F). Thus, we demonstrated that CRC-EVs have the capability to bind to αvβ3, while MFGE8-deficient CRC-EVs showed a marked decrease in their binding to αvβ3. It suggests that MFGE8 plays a role in facilitating CRC-EVs interaction with αvβ3.

### MFGE8 promoted macrophage efferocytosis through activation of αvβ3-STAT3 signaling pathway

**Fig. 6:**
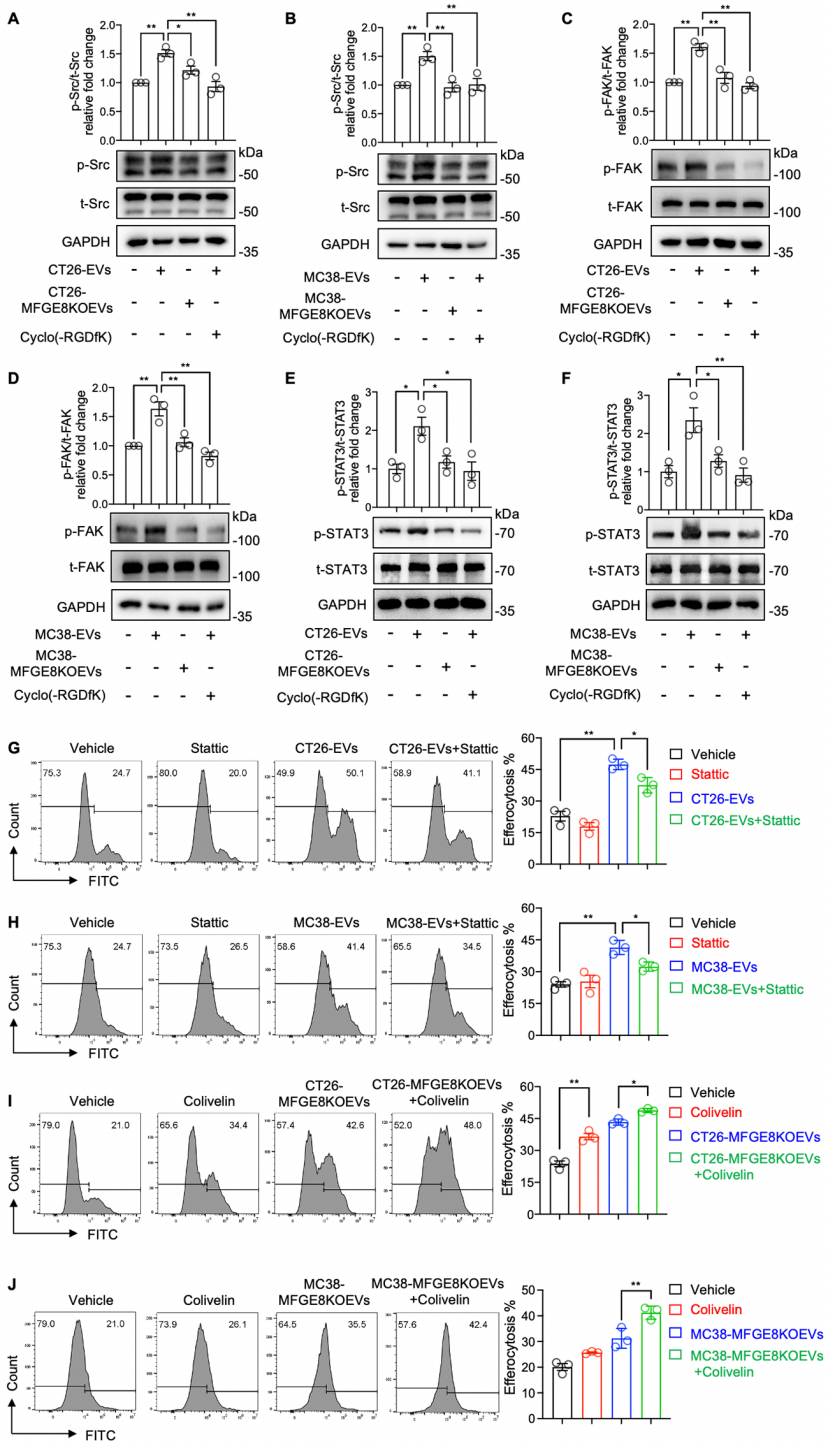
MFGE8 in CRC-EVs promoted macrophage efferocytosis through αVβ3-STAT3 signaling pathway. (**A** to **F**) BMDMs were treated with PBS (Control), 10 µg/ml CRC-EVs (CT26-EVs or MC38-EVs), 10 µg/ml MFGE8KOEVs, or 10 µg/ml CRC-EVs combined with 20 µM Cyclo(-RGDfK). The expression of phosphorylated Src (p-Src), total Src (t-Src), phosphorylated FAK (p-FAK), total FAK (t-FAK), phosphorylated STAT3 (p-STAT3), total STAT3 (t-STAT3) was measured by western blot. (**G** and **H**) BMDMs were treated with Vehicle (DMSO), 10 µM Stattic, 10 µg/ml CRC-EVs (CT26-EVs or MC38-EVs), or 10 µg/ml CRC-EVs combined with 10 µM Stattic (Stattic was added 30 min before EV treatment) for 24 h, followed by exposure to cisplatin-induced apoptotic tumor cells for 4 h. The efferocytosis percentage was analyzed by flow cytometry. (**I** and **J**) BMDMs were treated with Vehicle (DMSO), 20 µM Colivelin, 10 µg/ml CRC-EVs (CT26-EVs or MC38-EVs), or 10 µg/ml CRC-EVs combined with 20 µM Colivelin for 24 h, followed by exposure to cisplatin-induced apoptotic tumor cells for 4 h. The efferocytosis percentage was analyzed by flow cytometry. Data was shown in mean ± SEM of *n* = 3–4 independent experiments per condition. *P < 0.05 or **P < 0.01. One-way ANOVA with Tukey post-hoc comparisons

Previous studies have indicated that MFGE8 engages with macrophages by bridging integrin αvβ3 on their surface with phosphatidylserine (PS) present on apoptotic cells. This interaction leads to the phosphorylation and activation of Src and focal adhesion kinase (FAK). Activated FAK further triggers the activation of STAT3, facilitating cytoskeletal rearrangement, cell motility, and the formation of phagocytic vesicles, thereby completing the process of efferocytosis [39–41]. Therefore, we determined to investigate whether MFGE8 carried by CRC-EVs activates the Src-FAK-STAT3 signaling pathway through αvβ3 in macrophages. The results indicated that both CT26-EVs and MC38-EVs upregulated the phosphorylation of Src, FAK, and STAT3. Conversely, MFGE8-deficient EVs failed to induce phosphorylation of Src, FAK, and STAT3 (Fig. 6A and F). Additionally, treatment with Cyclo(-RGDfK), an inhibitor of αvβ3 integrin, effectively inhibited the phosphorylation and activation of Src, FAK, and STAT3 induced by CT26-EVs or MC38-EVs (Fig. 6A and F).

To confirm the role of STAT3 in mediating the efferocytosis effect of CRC-EVs on macrophages, we pre-treated BMDMs with Stattic, a phosphorylation inhibitor of STAT3, and found that it blocked the efferocytosis enhancement by CT26-EVs or MC38-EVs (Fig. 6G and H). To further confirm the role of STAT3 activation in promoting CRC-EVs-mediated macrophage efferocytosis, we treated MFGE8-knockout CRC-EVs (including CT26-MFGE8KOEVs and MC38-MFGE8KOEVs) with the STAT3 agonist Colivelin. As shown in Fig. 6I and J, there was a significant increase in the percentage of macrophage efferocytosis. These data suggest that MFGE8 in CRC-EVs activated the phosphorylation of STAT3 in macrophages through the αvβ3-Src-FAK signaling pathway.

### MFGE8 in CRC-EVs enhanced peritoneal macrophage efferocytosis

**Fig. 7:**
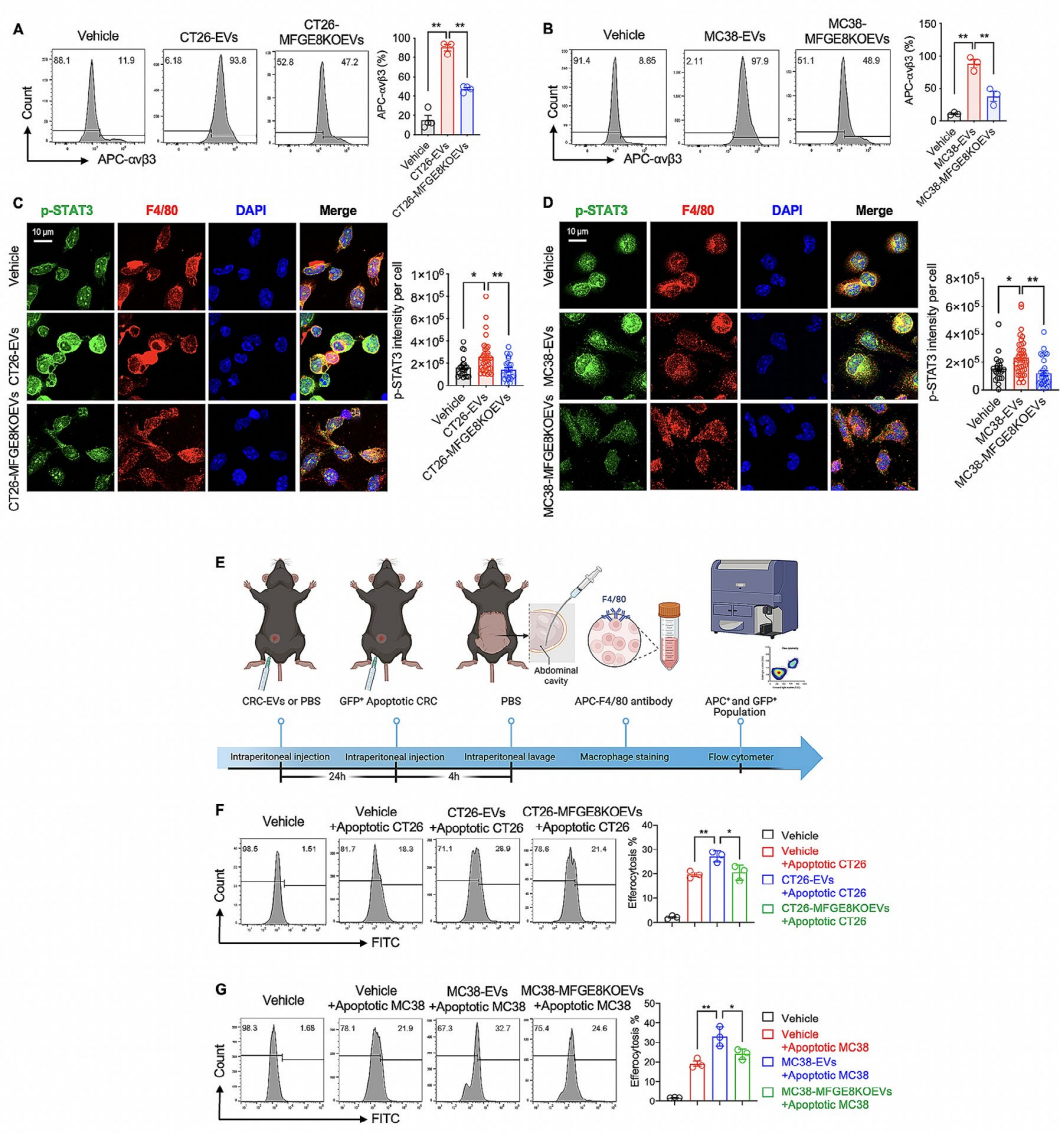
MFGE8 in CRC-EVs enhanced peritoneal macrophage efferocytosis. (**A-D**) 12-week-old C57BL/6 mice were intraperitoneally administered Vehicle (200 µl PBS), CT26-EVs (50 µg per mouse in 200 µl PBS), or CT26-MFGE8KOEVs (50 µg per mouse in 200 µl PBS) for 24 h. The animal experiment was divided into three groups, with each group representing an independent replicate experiment using one mouse per group. (**A-B**) Peritoneal cells were obtained from peritoneal lavage and stained with FITC-F4/80 and APC-αvβ3. Flow cytometry was employed to analyze the percentage of APC-αvβ3 positive population in the FITC-F4/80 positive peritoneal macrophage population. (**C-D**) Peritoneal cells were obtained from peritoneal lavage and stained with p-STAT3 and F4/80. Confocal microscopy was used to measure the intensity levels of p-STAT3 fluorescence in peritoneal macrophages expressing F4/80 fluorescence. All immunofluorescence images were acquired from three to four independent replicate experiments, with averages computed from three random fields for each experimental group. (**E**) Schematic illustration of how CRC-EVs enhance the efferocytosis of apoptotic tumor cells by peritoneal macrophages in mice. This experiment is divided into four groups, with one mouse per group, and a total of three independent repeated experiments are conducted. (**F**) C57BL/6 mice, aged 12 weeks, were intraperitoneally administered Vehicle (200 µl PBS), CT26-EVs (50 µg per mouse in 200 µl PBS), or CT26-MFGE8KOEVs (50 µg per mouse 200 µl PBS) for 24 h. After that, they were injected intraperitoneally with 2 × 10^6^ cisplatin-induced apoptotic GFP-CT26 cells for 4 h. The peritoneal cells were obtained from the peritoneal lavage and stained with APC-F4/80 antibody. The flow cytometry was used to analyze the proportion of peritoneal macrophages that efferocytosis of apoptotic GFP-CT26 cells (the percentage of GFP + cells in the APC + cell population). (**G**) C57BL/6 mice were intraperitoneally given Vehicle (200 µl PBS), MC38-EVs (50 µg per mouse in 200 µl PBS), or MC38-MFGE8KOEVs (50 µg per mouse in 200 µl PBS), followed by an intraperitoneal injection of cisplatin-induced apoptotic GFP-MC38 cells. The efferocytosis rate was determined by flow cytometry. Data was shown in mean ± SEM of *n* = 3–4 independent experiments per condition. *P < 0.05 or **P < 0.01. One-way ANOVA with Tukey post-hoc comparisons

The aforementioned in vitro experiments have provided comprehensive evidence that CRC-EVs containing MFGE8 enhance macrophage efferocytosis by activating the αvβ3-STAT3 signaling pathway. To further substantiate these findings, we conducted the in vivo animal experiments. Initially, we administered CRC-EVs (including CT26-EVs and MC38-EVs) via intraperitoneal injection to ascertain whether they could prompt activation of the macrophage αvβ3-STAT3 signaling pathway in vivo. As shown in Fig. 7A and D, we found that intraperitoneal injection of CRC-EVs led to an increase in the expression of αvβ3 (as indicated by the percentage of APC-αvβ3-positive population within FITC-F4/80-positive population analyzed through flow cytometry, Fig. 7A and B) and elevated phosphorylation levels of STAT3 (measured by the immunofluorescence intensity of p-STAT3 in F4/80 staining using confocal microscopy, Fig. 7C and D) in peritoneal macrophages of mice. Conversely, administration of CRC-MFGE8KOEVs (both CT26-MFGE8KOEVs and MC38-MFGE8KOEVs) failed to induce upregulation of αvβ3 and p-STAT3 levels. This consistent evidence suggests that in vivo, CRC-EVs containing MFGE8 activate the macrophage αvβ3-STAT3 signaling pathway (Fig. 7A and D). To further confirm the pro-efferocytosis effect of CRC-EVs in vivo, the mice were given an intraperitoneal injection of either CRC-EVs or PBS (Vehicle) for 24 h, followed by an intraperitoneal injection of apoptotic CRC cells for 4 h (Fig. 7E). We then collected peritoneal lavage cells and stained them with APC-F4/80 to identify the peritoneal macrophage population (Fig. 7E). As seen in Fig. 7F, compared to the Vehicle group, the administration of CT26-EVs significantly increased the efferocytosis of cisplatin-induced apoptotic CT26 cells by peritoneal macrophages. In addition, the efferocytosis percentage of apoptotic cells in the CT26-MFGE8KOEVs treatment group was significantly lower compared to the CT26-EVs treatment group (Fig. 7F). The same result was found in the treatment of MC38-EVs, where the peritoneal macrophage efferocytosis of cisplatin-induced apoptotic cells was enhanced, but the effect was diminished in the absence of MFGE8 in MC38-EVs (Fig. 7G). These results suggest that CRC-EVs enhance the efferocytotic ability of peritoneal macrophages in vivo and that the pro-efferocytosis effect is due to the presence of MFGE8 in CRC-EVs. These in vivo results are consistent with our in vitro findings.

## Discussion

It is well known that the cytotoxic effects of chemotherapy induce apoptosis in CRC cells, leading to the release of a significant amount of pro-inflammatory factors. Macrophages play a crucial role in engulfing apoptotic cells and maintaining tissue balance [42]. These approaching macrophages recognize the chemoattractants, such as lipids, proteins, and peptides, that are released by apoptotic cells [43]. The “eat-me” signals in apoptotic cells are identified by the efferocytosis-related receptors on macrophages, enabling them to carry out their phagocytic function [43]. This leads to cytoskeletal rearrangement in phagocytes, cell mobilization, and the formation of phagosomes to complete the phagocytic process [44]. Efferocytosis is accompanied by the secretion of anti-inflammatory cytokines such as TGF-β, IL-10, and PGE2, while the production of pro-inflammatory cytokines such as IL-1β, TNF-α, and IL-12 is suppressed [19]. It is worth noting that efferocytosis promotes the polarization of macrophages towards the tumor-promoting M2 phenotype [36]. Efferocytosis-related receptors, such as MerTK and AXL, have been found to play a role in promoting tumor progression. Such as Yan et al. discovered that MerTK activation was responsible for osimertinib resistance in EGFR-mutant non-small cell lung cancer [45]. Lin et al. reported that MerTK-mediated efferocytosis promoted immune tolerance and tumor progression in osteosarcoma by enhancing M2 polarization and PD-L1 expression [25]. Zdżalik-Bielecka et al. found that the GAS6-AXL signaling pathway triggered actin remodeling that drove membrane ruffling, macropinocytosis, and cancer-cell invasion [46]. In conclusion, efferocytosis dampens chemotherapy-induced inflammatory responses, hastens injury healing, and promotes tumor progression.

In the tumor microenvironment (TME), macrophages constitute one of the most abundant immune cell types. Within solid tumors, cell death is a prevalent occurrence that escalates with cytotoxic treatments like cisplatin. The process of macrophages engulfing and clearing dead cells, known as efferocytosis, exerts an immunosuppressive influence. Conversely, the uncleared apoptotic cells can promote anti-tumor immunity [47]. As efferocytosis progresses, macrophages adopt an M2-like phenotype [48]. This transition prompts the production of immunosuppressive cytokines such as IL-4, IL-10, IL-13, and TGF-β, which recruit FOXP3 + regulatory T cells, effectively suppressing the effector functions of CD4 + and CD8 + T cells within the TME [49]. Consequently, efferocytosis contributes to immune tolerance in the TME, thus facilitating tumor progression. However, the role of macrophages in tumors may exhibit dual characteristics. Studies have shown that M1 polarization in CRC promotes the expression of galectin-3, enhancing macrophage infiltration and sensitizing immune responses against tumor cells [50]. Furthermore, M1 macrophage polarization induces the production of pro-inflammatory cytokines such as TNF, IFN, and IL-1 in the TME of CRC, recruiting cytotoxic immune cells like CD8 + T cells and natural killer cells, thereby exerting anti-tumor effects [51]. Recently, we have known that extracellular vesicles (EVs) as a crucial intercellular communicator in TME [52]. Despite macrophages primarily assuming an immunosuppressive role, EVs derived from macrophages exhibit similar anti-tumor properties to their parent cells. For instance, CRC-EVs carrying miRNA induce macrophage M2 polarization via activation of the PI3K/Akt signaling pathway [53]. Moreover, CRC-EVs induce programmed death-ligand 1 (PD-L1) expression in tumor-associated macrophages, facilitating tumor evasion [54]. Additionally, EVs from CRC stem cells promote tumor initiation by upregulating IL-1β expression to enhance neutrophil survival [55]. These findings underscore the regulatory impact of CRC-EVs on immune cells within the TEM, thereby exerting pro-tumorigenic effects.

An increasing number of studies are showing that EVs have pro-efferocytosis and anti-inflammatory effects in various diseases. For instance, Mentkowski et al. found that EVs from cardiosphere-derived cells enhanced efferocytosis and stimulated Arg1-dependent angiogenesis, making them an immunomodulatory and cardioprotective factor in cardiovascular disease [56]. Similarly, Gomez-Ferrer et al. found that EVs from HIF-overexpressed mesenchymal stem cells increased efferocytotic capacity in Crohn’s disease [57]. Zhang et al. demonstrated that exosomal miR-16 and miR-21 from bone marrow-derived mesenchymal stem cells helped alleviate systemic lupus erythematosus by promoting efferocytosis [58]. However, there is limited study focused on how tumor-derived EVs affect macrophage efferocytosis and then consequently influence tumor progression. Wolf-Dennen et al. reported that EVs from metastatic osteosarcoma decreased efferocytosis in alveolar macrophages [59]. Our previous study has shown that CRC-EVs containing HSP70 enhance macrophage phagocytosis by upregulating MARCO expression [60]. In this study, we found that EVs isolated from CRC (including CT26 and MC38) cells enhanced macrophage efferocytosis, which is different from the findings of Wolf-Dennen et al. This discrepancy might be attributed to variations in the tumor type and the conditions under which the EVs were isolated.

Efferocytosis, the process of engulfing and clearing apoptotic cells, relies on the recognition of “eat me” signals by engulfing receptors or through bridging molecules such as MFGE8 [61]. MFGE8 serves as a crucial link between apoptotic cells and phagocytes, as demonstrated in a study by Kranich et al. where MFGE8 secreted by follicular dendritic cells promoted macrophage clearance of apoptotic B cells, while MFGE8 deletion resulted in impaired clearance of apoptotic cells and accumulation of apoptotic cell debris in germinal centers [62]. Another study by Fens et al. showed that MFGE8 induced angiogenic endothelial cells in tumors to phagocytose apoptotic melanoma cells [63]. Soki et al. reported that MFGE8 from prostate cancer promoted macrophage efferocytosis and also induced an up-regulation of MFGE8 in macrophages undergoing efferocytosis [36]. Additionally, a recent study on myocardial infarction revealed that exosomal delivery of MFGE8 from mesenchymal stem cells played an opsonizing effect on apoptotic cells and enhanced the efferocytotic activity of macrophages [38]. Zhao et al. have demonstrated that poorer prognosis in colorectal cancer patients with high MFGE8 expression. Additionally, the effect of MFGE8 on colorectal cancer cell migration, invasion, and epithelial-to-mesenchymal transition was found to be partially dependent on the PI3K/AKT signaling pathway [33]. Another study by Jia et al. indicated a significant increase in MFGE8 expression in colorectal cancer compared to normal mucosa tissues, with patients exhibiting high levels of MFGE8 protein showing shortened overall survival [32]. Both clinical studies underscored the upregulation of MFGE8 expression in colorectal cancer tumor tissues, and patients with high MFGE8 expression had poorer prognoses. This is consistent with the findings of our study, which further suggests that MFGE8 carried in CRC-EVs may exacerbate the prognosis of CRC by enhancing cisplatin resistance through macrophage efferocytosis.

In our study, we conducted a mass spectrometry-based proteomic analysis of CT26-EVs and found that MFGE8 had a high abundance. This finding was confirmed by immunoblotting, which showed high expression of MFGE8 in CRC-EVs. We then used the CRISPR-Cas9 system to create MFGE8-knockout CRC (CT26 and MC38) cells, resulting in MFGE8-deficient CRC-EVs. Compared to wild-type CRC-EVs, the MFGE8-deficient CRC-EVs did not effectively activate BMDMs to efferocytosis of apoptotic tumor cells, a result that was replicated in vivo. MFGE8 knockout significantly reduced CRC-EVs-mediated efferocytosis in peritoneal macrophages. To understand the molecular mechanism behind CRC-EVs regulation of macrophage efferocytosis, we focused on the αvβ3-STAT3 signaling pathway. Ren et al. reported that the αvβ3-FAK-STAT3 pathway was responsible for activating efferocytosis in acute pancreatitis [39]. In our study, we found that MFGE8 carried within CRC-EVs up-regulated the expression of cell surface αvβ3 on macrophages. Subsequent blockade of the αvβ3 receptor on macrophages using Cyclo(-RGDfK) effectively inhibited CRC-EVs-induced phosphorylation of Src, FAK, and STAT3 in macrophages. This inhibition further prevented CRC-EVs-induced efferocytosis in macrophages. The application of Stattic, a specific inhibitor of STAT3 phosphorylation, also hindered the promotion of macrophage efferocytosis by CRC-EVs, indicating that phosphorylated STAT3 mediates this efferocytic process. In addition, CRC-EVs lacking MFGE8 failed to activate the αvβ3-Src-FAK-STAT3 signaling pathway in macrophages. However, treatment with Colivelin, a STAT3 activator, restored the efferocytic function of macrophages, highlighting the pivotal role of phosphorylated STAT3 in mediating efferocytosis.

It is widely recognized that cisplatin, a type of chemotherapy drug, can cause tumor cell death through DNA damage [64]. Cisplatin also has anti-tumor immune effects, such as increasing the expression of MHC Class I molecules, reducing Treg lymphocytes, and attracting immune cells [64]. Although cisplatin has proven successful in treating various tumor entities, CRC has been shown to be unresponsive to cisplatin, limiting the success of treatment [65]. Resistance to cisplatin is frequently observed in tumor cells, and this resistance may be intrinsic to certain tumors, such as pancreatic and colorectal cancer, or it may develop during the course of treatment, as seen in ovarian cancer [65]. The presence of cisplatin resistance poses a significant challenge in achieving therapeutic success in CRC. Understanding the mechanisms underlying this resistance is crucial for developing strategies to overcome or circumvent it, thereby improving the efficacy of cisplatin-based treatments in CRC. In this study, we elucidated a novel molecular mechanism of cisplatin resistance in CRC. This mechanism involves non-apoptotic CRC cells delivering MFGE8 through EVs to macrophages. Subsequently, this process activates macrophage efferocytosis, leading to the clearance of apoptotic tumor cells induced by cisplatin. Therefore, this study has revealed a novel mechanism by which CRC exhibits resistance to cisplatin treatment through the secretion of EVs.

## Conclusions

**Fig. 8:**
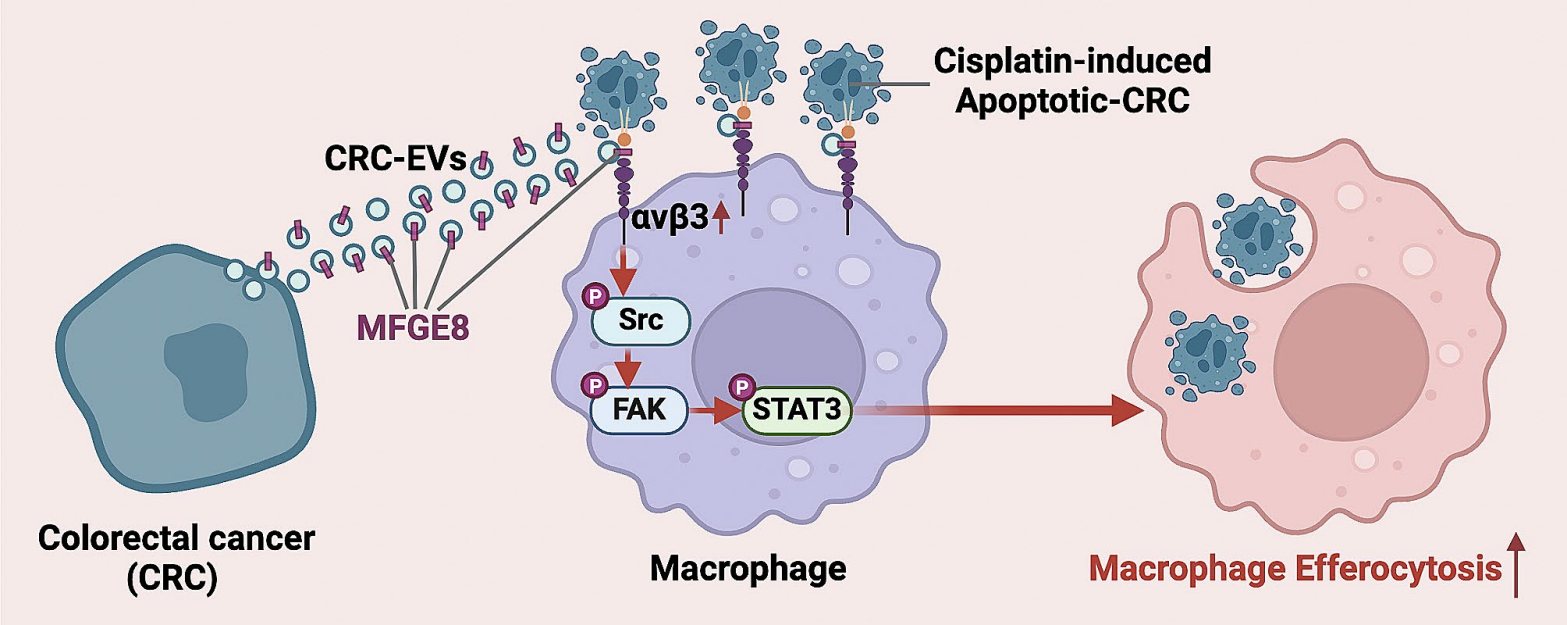
Schematic model of CRC-EVs induced macrophage efferocytosis. In the process of treating colorectal cancer (CRC) with cisplatin, cisplatin can induce apoptosis in CRC cells. CRC cells that have not undergone apoptosis secrete EVs containing the MFGE8 protein, which are delivered to macrophages. Upon delivery, MFGE8 upregulates αvβ3 cell surface expression and subsequently activates the Src-FAK-STAT3 signaling pathway in macrophages. This activation promotes efferocytosis, facilitating the clearance of apoptotic tumor cells induced by cisplatin. Ultimately, this resistance to the therapeutic effects of cisplatin promotes the progression of CRC tumors

In conclusion, our study highlights the pro-efferocytosis role of MFGE8 in CRC-EVs and demonstrates that MFGE8 facilitates macrophage efferocytosis through the αvβ3-Src-FAK-STAT3 signaling pathway (Fig. 8). This provides new insight into the regulation of macrophage function during cisplatin chemotherapy by CRC-EVs. The combination of chemotherapy and efferocytotic inhibition may be a promising approach for CRC treatment.

### Electronic supplementary material

Below is the link to the electronic supplementary material.

Supplementary Material 1 - Supplementary Figures

Supplementary Material 2 - CT26-EVs MS Proteomic Data

Supplementary Material 3 - CRC patients MFGE8

Supplementary Material 4 - Uncropped Western Blot

## Data Availability

The data that support the findings of this study are available from the corresponding author upon reasonable request.

## Abbreviations

CRC: Colorectal cancer
EVs: Extracellular vesicles
TME: Tumor microenvironment
TAMs: Tumor-associated macrophages
BMDMs: Bone marrow-derived macrophages
MFGE8: Milk fat globule-epidermal growth factor-factor 8
GFP: Green fluorescent protein
CRC-EVs: Colorectal cancer-derived EVs
CT26-EVs: CT26 cells-derived EVs
CT26-MFGE8KOEVs: EVs derived from MFGE8-knockout CT26 cells
GFP-CT26: CT26 cells stably expressing green fluorescent protein
MC38-EVs: MC38 cells-derived EVs
MC38-MFGE8KOEVs: EVs derived from MFGE8-knockout MC38 cells
GFP-MC38: MC38 cells stably expressing green fluorescent protein
FAK: Focal adhesion kinase

## References

[1] Siegel RL, Miller KD, Wagle NS, Jemal A (2023). Cancer statistics, 2023. CA Cancer J Clin.

[2] Siegel RL, Miller KD, Goding Sauer A, Fedewa SA, Butterly LF, Anderson JC, Cercek A, Smith RA, Jemal A (2020). Colorectal cancer statistics, 2020. CA Cancer J Clin.

[3] Koberle B, Schoch S. Platinum complexes in Colorectal Cancer and other solid tumors. Cancers (Basel) 2021, 13. 10.3390/cancers13092073 PMC8123298 33922989

[4] Fan WH, Wang FC, Jin Z, Zhu L, Zhang JX (2022). Curcumin synergizes with Cisplatin to inhibit Colon cancer through targeting the MicroRNA-137-Glutaminase Axis. Curr Med Sci.

[5] Wang Y, Lina L, Xu L, Yang Z, Qian Z, Zhou J, Suoni L (2019). Arctigenin enhances the sensitivity of cisplatin resistant colorectal cancer cell by activating autophagy. Biochem Biophys Res Commun.

[6] Zhang P, Zhao S, Lu X, Shi Z, Liu H, Zhu B (2020). Metformin enhances the sensitivity of colorectal cancer cells to cisplatin through ROS-mediated PI3K/Akt signaling pathway. Gene.

[7] Chen G, Huang AC, Zhang W, Zhang G, Wu M, Xu W, Yu Z, Yang J, Wang B, Sun H (2018). Exosomal PD-L1 contributes to immunosuppression and is associated with anti-PD-1 response. Nature.

[8] Yáñez-Mó M, Siljander PR, Andreu Z, Zavec AB, Borràs FE, Buzas EI, Buzas K, Casal E, Cappello F, Carvalho J (2015). Biological properties of extracellular vesicles and their physiological functions. J Extracell Vesicles.

[9] Buzas EI. The roles of extracellular vesicles in the immune system. Nat Rev Immunol 2022:1–15. 10.1038/s41577-022-00763-8 PMC9361922 35927511

[10] Kalluri R, LeBleu VS. The biology, function, and biomedical applications of exosomes. Science 2020, 367. 10.1126/science.aau6977 PMC7717626 32029601

[11] van Niel G, D’Angelo G, Raposo G (2018). Shedding light on the cell biology of extracellular vesicles. Nat Rev Mol Cell Biol.

[12] Zhao S, Mi Y, Guan B, Zheng B, Wei P, Gu Y, Zhang Z, Cai S, Xu Y, Li X (2020). Tumor-derived exosomal miR-934 induces macrophage M2 polarization to promote liver metastasis of colorectal cancer. J Hematol Oncol.

[13] Liu J, Fan L, Yu H, Zhang J, He Y, Feng D, Wang F, Li X, Liu Q, Li Y (2019). Endoplasmic reticulum stress causes Liver Cancer cells to Release Exosomal miR-23a-3p and Up-regulate programmed death Ligand 1 expression in macrophages. Hepatology.

[14] Liang M, Chen X, Wang L, Qin L, Wang H, Sun Z, Zhao W, Geng B (2020). Cancer-derived exosomal TRIM59 regulates macrophage NLRP3 inflammasome activation to promote lung cancer progression. J Exp Clin Cancer Res.

[15] Lan J, Sun L, Xu F, Liu L, Hu F, Song D, Hou Z, Wu W, Luo X, Wang J (2019). M2 macrophage-derived Exosomes Promote Cell Migration and Invasion in Colon cancer. Cancer Res.

[16] Zheng P, Chen L, Yuan X, Luo Q, Liu Y, Xie G, Ma Y, Shen L (2017). Exosomal transfer of tumor-associated macrophage-derived miR-21 confers cisplatin resistance in gastric cancer cells. J Exp Clin Cancer Res.

[17] Yang Y, Guo Z, Chen W, Wang X, Cao M, Han X, Zhang K, Teng B, Cao J, Wu W (2021). M2 macrophage-derived exosomes promote angiogenesis and growth of pancreatic ductal adenocarcinoma by targeting E2F2. Mol Ther.

[18] Vitale I, Manic G, Coussens LM, Kroemer G, Galluzzi L (2019). Macrophages and metabolism in the Tumor Microenvironment. Cell Metab.

[19] Mehrotra P, Ravichandran KS (2022). Drugging the efferocytosis process: concepts and opportunities. Nat Rev Drug Discov.

[20] Moon B, Lee J, Lee SA, Min C, Moon H, Kim D, Yang S, Moon H, Jeon J, Joo YE, Park D. Mertk interacts with Tim-4 to enhance Tim-4-Mediated Efferocytosis. Cells 2020, 9. 10.3390/cells9071625 PMC7408610 32640697

[21] Tang J, Jin Y, Jia F, Lv T, Manaenko A, Zhang LF, Zhang Z, Qi X, Xue Y, Zhao B et al. Gas6 promotes Microglia Efferocytosis and suppresses inflammation through activating Axl/Rac1 signaling in Subarachnoid Hemorrhage mice. Transl Stroke Res 2022. 10.1007/s12975-022-01099-0 36324028

[22] Zhou Y, Yao Y, Deng Y, Shao A (2020). Regulation of efferocytosis as a novel cancer therapy. Cell Commun Signal.

[23] Roca H, Jones JD, Purica MC, Weidner S, Koh AJ, Kuo R, Wilkinson JE, Wang Y, Daignault-Newton S, Pienta KJ (2018). Apoptosis-induced CXCL5 accelerates inflammation and growth of prostate tumor metastases in bone. J Clin Invest.

[24] Yang M, Liu J, Piao C, Shao J, Du J (2015). ICAM-1 suppresses tumor metastasis by inhibiting macrophage M2 polarization through blockade of efferocytosis. Cell Death Dis.

[25] Lin J, Xu A, Jin J, Zhang M, Lou J, Qian C, Zhu J, Wang Y, Yang Z, Li X (2022). MerTK-mediated efferocytosis promotes immune tolerance and tumor progression in osteosarcoma through enhancing M2 polarization and PD-L1 expression. Oncoimmunology.

[26] Cheng L, Weng B, Jia C, Zhang L, Hu B, Deng L, Mou N, Sun F, Hu J (2022). The expression and significance of efferocytosis and immune checkpoint related molecules in pancancer samples and the correlation of their expression with anticancer drug sensitivity. Front Pharmacol.

[27] Akakura S, Singh S, Spataro M, Akakura R, Kim JI, Albert ML, Birge RB (2004). The opsonin MFG-E8 is a ligand for the alphavbeta5 integrin and triggers DOCK180-dependent Rac1 activation for the phagocytosis of apoptotic cells. Exp Cell Res.

[28] Hanayama R, Tanaka M, Miwa K, Shinohara A, Iwamatsu A, Nagata S (2002). Identification of a factor that links apoptotic cells to phagocytes. Nature.

[29] Lemke G (2019). How macrophages deal with death. Nat Rev Immunol.

[30] Motegi S, Leitner WW, Lu M, Tada Y, Sárdy M, Wu C, Chavakis T, Udey MC (2011). Pericyte-derived MFG-E8 regulates pathologic angiogenesis. Arterioscler Thromb Vasc Biol.

[31] Bu HF, Zuo XL, Wang X, Ensslin MA, Koti V, Hsueh W, Raymond AS, Shur BD, Tan XD (2007). Milk fat globule-EGF factor 8/lactadherin plays a crucial role in maintenance and repair of murine intestinal epithelium. J Clin Invest.

[32] Jia M, Yao H, Chen C, Wang Y, Wang H, Cui T, Zhu J (2017). Prognostic correlation between MFG-E8 expression level and colorectal Cancer. Arch Med Res.

[33] Zhao Q, Xu L, Sun X, Zhang K, Shen H, Tian Y, Sun F, Li Y (2017). MFG-E8 overexpression promotes colorectal cancer progression via AKT/MMPs signalling. Tumour Biol.

[34] Li Z, Scott MJ, Fan EK, Li Y, Liu J, Xiao G, Li S, Billiar TR, Wilson MA, Jiang Y, Fan J (2016). Tissue damage negatively regulates LPS-induced macrophage necroptosis. Cell Death Differ.

[35] Li Z, Moniruzzaman M, Dastgheyb RM, Yoo SW, Wang M, Hao H, Liu J, Casaccia P, Nogueras-Ortiz C, Kapogiannis D (2020). Astrocytes deliver CK1 to neurons via extracellular vesicles in response to inflammation promoting the translation and amyloidogenic processing of APP. J Extracell Vesicles.

[36] Soki FN, Koh AJ, Jones JD, Kim YW, Dai J, Keller ET, Pienta KJ, Atabai K, Roca H, McCauley LK (2014). Polarization of prostate cancer-associated macrophages is induced by milk fat globule-EGF factor 8 (MFG-E8)-mediated efferocytosis. J Biol Chem.

[37] Xiang X, Zhao X, Pan X, Dong Z, Yu J, Li S, Liang X, Han P, Qu K, Jensen JB (2021). Efficient correction of Duchenne muscular dystrophy mutations by SpCas9 and dual gRNAs. Mol Ther Nucleic Acids.

[38] Patil M, Saheera S, Dubey PK, Kahn-Krell A, Kumar Govindappa P, Singh S, Tousif S, Zhang Q, Lal H, Zhang J (2021). Novel mechanisms of exosome-mediated phagocytosis of dead cells in injured heart. Circ Res.

[39] Ren Y, Liu W, Zhang L, Zhang J, Bi J, Wang T, Wang M, Du Z, Wang Y, Zhang L (2021). Milk fat globule EGF factor 8 restores mitochondrial function via integrin-medicated activation of the FAK-STAT3 signaling pathway in acute pancreatitis. Clin Transl Med.

[40] Schaller MD (2010). Cellular functions of FAK kinases: insight into molecular mechanisms and novel functions. J Cell Sci.

[41] Serrels B, Serrels A, Brunton VG, Holt M, McLean GW, Gray CH, Jones GE, Frame MC (2007). Focal adhesion kinase controls actin assembly via a FERM-mediated interaction with the Arp2/3 complex. Nat Cell Biol.

[42] Doran AC, Yurdagul A, Tabas I (2020). Efferocytosis in health and disease. Nat Rev Immunol.

[43] Boada-Romero E, Martinez J, Heckmann BL, Green DR (2020). The clearance of dead cells by efferocytosis. Nat Rev Mol Cell Biol.

[44] Yin C, Heit B (2021). Cellular responses to the efferocytosis of apoptotic cells. Front Immunol.

[45] Yan D, Huelse JM, Kireev D, Tan Z, Chen L, Goyal S, Wang X, Frye SV, Behera M, Schneider F et al. MERTK activation drives osimertinib resistance in EGFR-mutant non-small cell lung cancer. J Clin Invest 2022, 132. 10.1172/JCI150517 PMC9337831 35708914

[46] Zdżalik-Bielecka D, Poświata A, Kozik K, Jastrzębski K, Schink KO, Brewińska-Olchowik M, Piwocka K, Stenmark H, Miączyńska M. The GAS6-AXL signaling pathway triggers actin remodeling that drives membrane ruffling, macropinocytosis, and cancer-cell invasion. Proc Natl Acad Sci U S A 2021, 118. 10.1073/pnas.2024596118 PMC8285903 34244439

[47] Poon IK, Lucas CD, Rossi AG, Ravichandran KSJNRI. Apoptotic cell clearance: basic biology and therapeutic potential. 2014, 14:166–80. 10.1038/nri3607 PMC4040260 24481336

[48] Lin J, Xu A, Jin J, Zhang M, Lou J, Qian C, Zhu J, Wang Y, Yang Z, Li XJO. MerTK-mediated efferocytosis promotes immune tolerance and tumor progression in osteosarcoma through enhancing M2 polarization and PD-L1 expression. 2022, 11:2024941. 10.1080/2162402X.2021.2024941 PMC8757471 35036076

[49] Guruvayoorappan CJIct. Tumor versus tumor-associated macrophages: how hot is the link? 2008, 7:90–5. 10.1177/1534735408319060 18550889

[50] Dumont P, Berton A, Nagy N, Sandras F, Tinton S, Demetter P, Mascart F, Allaoui A, Decaestecker C. Salmon IJLi: expression of galectin-3 in the tumor immune response in colon cancer. 2008, 88:896–906. 10.1038/labinvest.2008.54 18542048

[51] Ong SM, Tan YC, Beretta O, Jiang D, Yeap WH, Tai JJ, Wong WC, Yang H, Schwarz H. Lim KHJEjoi: macrophages in human colorectal cancer are pro-inflammatory and prime T cells towards an anti‐tumour type‐1 inflammatory response. 2012, 42:89–100. 10.1002/eji.201141825 22009685

[52] Siveen KS, Raza A, Ahmed EI, Khan AQ, Prabhu KS, Kuttikrishnan S, Mateo JM, Zayed H, Rasul K, Azizi F et al. The role of Extracellular vesicles as modulators of the Tumor Microenvironment, Metastasis and Drug Resistance in Colorectal Cancer. Cancers (Basel) 2019, 11. 10.3390/cancers11060746 PMC6628238 31146452

[53] Wang D, Wang X, Si M, Yang J, Sun S, Wu H, Cui S, Qu X, Yu X (2020). Exosome-encapsulated miRNAs contribute to CXCL12/CXCR4-induced liver metastasis of colorectal cancer by enhancing M2 polarization of macrophages. Cancer Lett.

[54] Yin Y, Liu B, Cao Y, Yao S, Liu Y, Jin G, Qin Y, Chen Y, Cui K, Zhou L (2022). Colorectal Cancer-derived small extracellular vesicles promote Tumor Immune Evasion by upregulating PD-L1 expression in Tumor-Associated macrophages. Adv Sci (Weinh).

[55] Hwang WL, Lan HY, Cheng WC, Huang SC, Yang MH (2019). Tumor stem-like cell-derived exosomal RNAs prime neutrophils for facilitating tumorigenesis of colon cancer. J Hematol Oncol.

[56] Mentkowski KI, Mursleen A, Snitzer JD, Euscher LM, Lang JK (2020). CDC-derived extracellular vesicles reprogram inflammatory macrophages to an arginase 1-dependent proangiogenic phenotype. Am J Physiol Heart Circ Physiol.

[57] Gómez-Ferrer M, Amaro-Prellezo E, Dorronsoro A, Sánchez-Sánchez R, Vicente Á, Cosín-Roger J, Barrachina MD, Baquero MC, Valencia J, Sepúlveda P. HIF-Overexpression and Pro-Inflammatory Priming in Human Mesenchymal Stromal Cells Improves the Healing Properties of Extracellular Vesicles in Experimental Crohn’s Disease. Int J Mol Sci 2021, 22. 10.3390/ijms222011269 PMC8540690 34681929

[58] Zhang M, Johnson-Stephenson TK, Wang W, Wang Y, Li J, Li L, Zen K, Chen X, Zhu D (2022). Mesenchymal stem cell-derived exosome-educated macrophages alleviate systemic lupus erythematosus by promoting efferocytosis and recruitment of IL-17(+) regulatory T cell. Stem Cell Res Ther.

[59] Wolf-Dennen K, Gordon N, Kleinerman ES (2020). Exosomal communication by metastatic osteosarcoma cells modulates alveolar macrophages to an M2 tumor-promoting phenotype and inhibits tumoricidal functions. Oncoimmunology.

[60] Sun Y, Xiao W, Yu Y, Jiang Y, Xiao Z, Huang D, Zhong T, Li J, Xiang X, He Y, Li Z (2023). Colorectal cancer-derived extracellular vesicles containing HSP70 enhance macrophage phagocytosis by up-regulating MARCO expression. Exp Cell Res.

[61] Li BZ, Zhang HY, Pan HF, Ye DQ (2013). Identification of MFG-E8 as a novel therapeutic target for diseases. Expert Opin Ther Targets.

[62] Kranich J, Krautler NJ, Heinen E, Polymenidou M, Bridel C, Schildknecht A, Huber C, Kosco-Vilbois MH, Zinkernagel R, Miele G, Aguzzi A (2008). Follicular dendritic cells control engulfment of apoptotic bodies by secreting Mfge8. J Exp Med.

[63] Fens MH, Mastrobattista E, de Graaff AM, Flesch FM, Ultee A, Rasmussen JT, Molema G, Storm G, Schiffelers RM (2008). Angiogenic endothelium shows lactadherin-dependent phagocytosis of aged erythrocytes and apoptotic cells. Blood.

[64] Dasari S, Tchounwou PB (2014). Cisplatin in cancer therapy: molecular mechanisms of action. Eur J Pharmacol.

[65] Kelland L (2007). The resurgence of platinum-based cancer chemotherapy. Nat Rev Cancer.

