# Supplementary material for: Extracellular vesicles containing MFGE8 from colorectal cancer facilitate macrophage efferocytosis
Source: Cell Commun Signal. 2024 May 27;22:295. doi: 10.1186/s12964-024-01669-9 (PMC11131254; doi:10.1186/s12964-024-01669-9)
Supplement: Supplementary file 1 — Supplementary Material 1 - Supplementary Figures [file 12964_2024_1669_MOESM1_ESM.docx]

**Supplementary Figures**

**
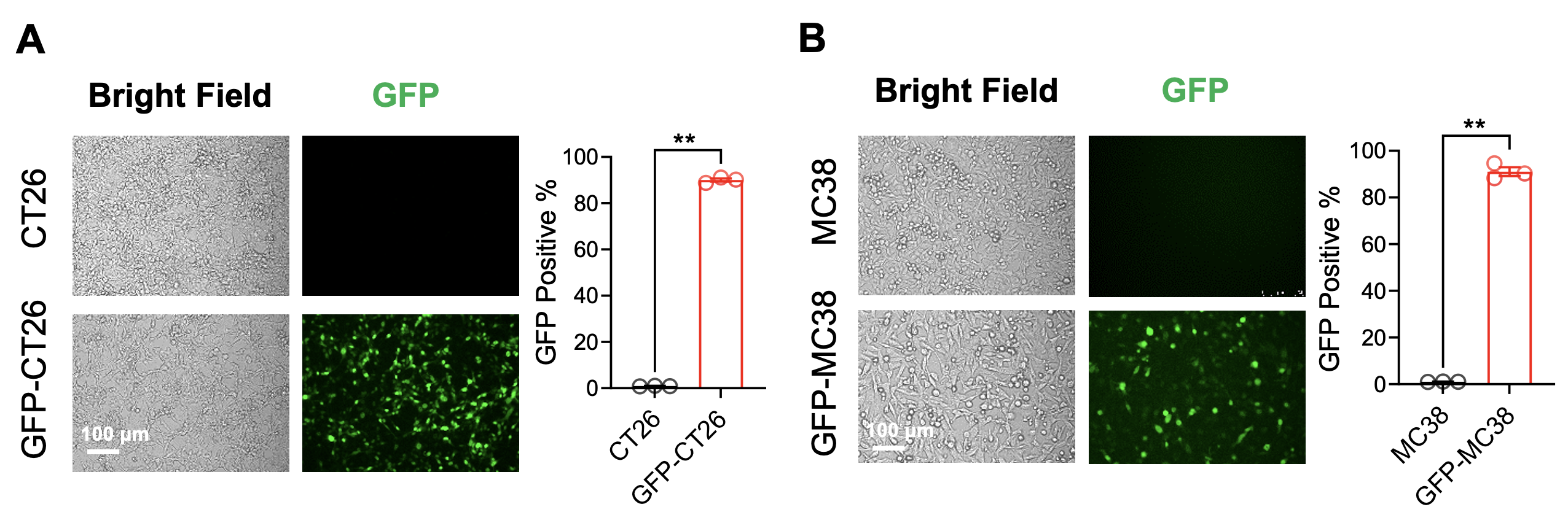
**

**Figure S1. Identification of GFP-CT26 and GFP-MC38 cells.** (A) Fluorescence microscopy images of CT26 and GFP-CT26 cells demonstrating the bright field and GFP fluorescence expression. (B) Fluorescence microscopy images of MC38 and GFP-MC38 cells displaying the bright field and GFP fluorescence. The percentage of cells expressing GFP was calculated using LEICA fluorescence microscopy. Data was shown in mean ± SEM of n=3 independent experiments per condition. ***P*<0.01. Student’s t-test.

**
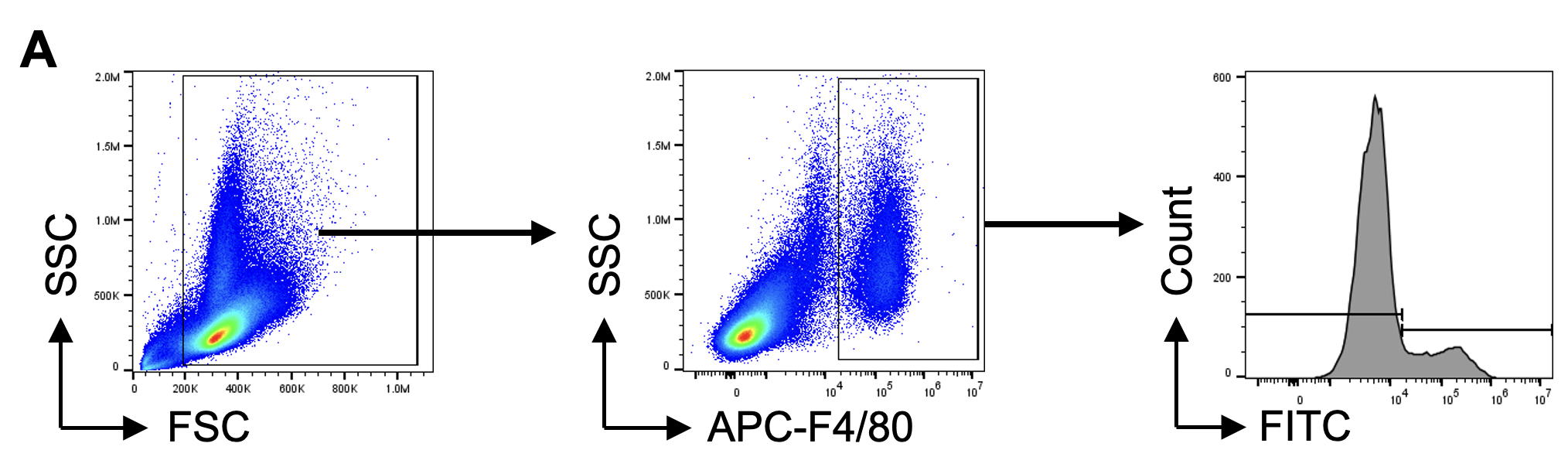
**

**Figure S2. Flow cytometry gating strategy.** (A) The rate of efferocytosis was calculated by determining the percentage of bone marrow-derived macrophages (BMDMs) (stained using an APC-F4/80 antibody, a marker for macrophages) that had efferocytosis of cisplatin-induced apoptotic GFP-overexpressed tumor cells (GFP+ population in the APC-F4/80+ population).

**
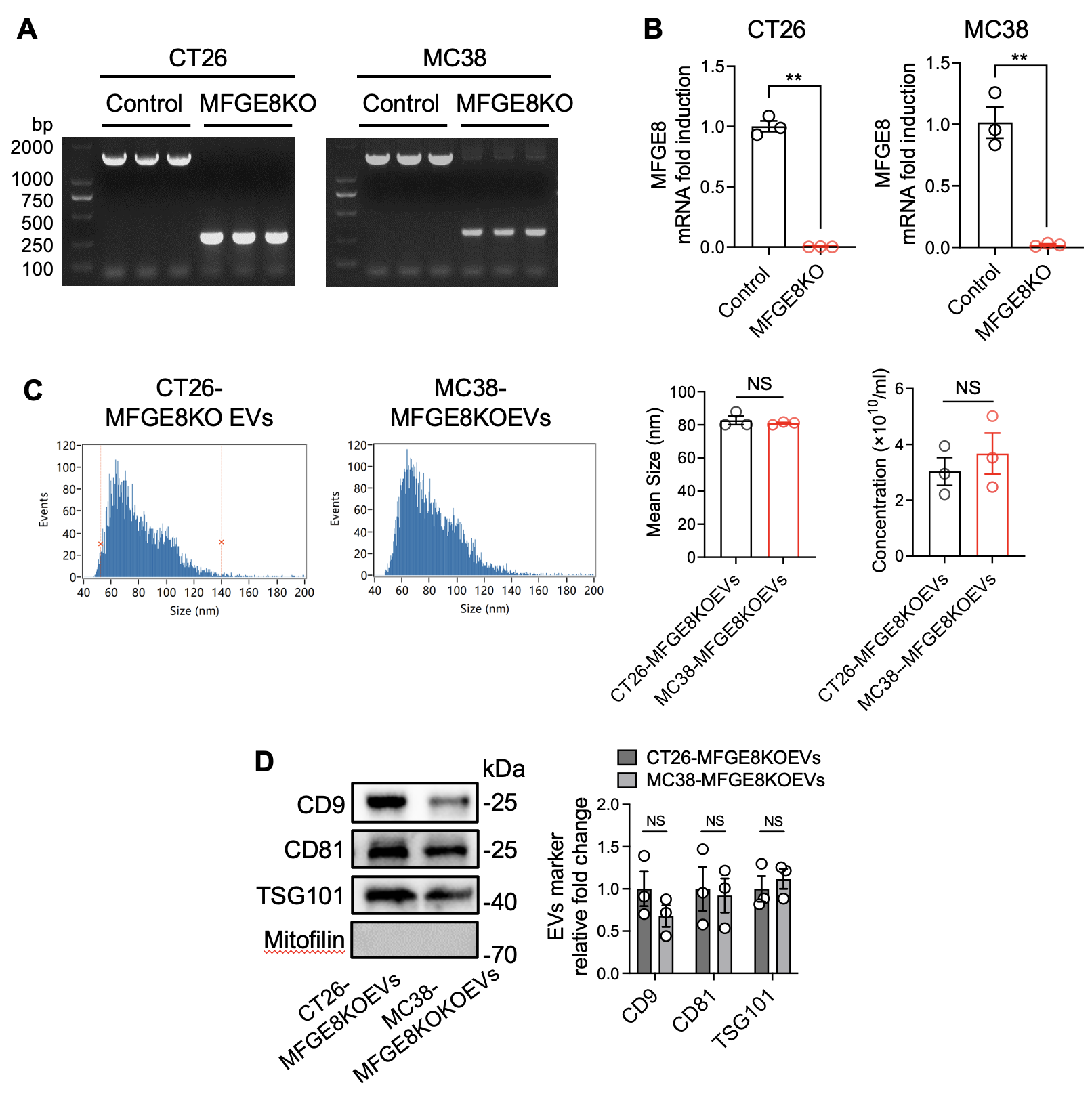
**

**Figure S3. Identification of MFGE8-knockout CRC-derived EVs.** (A) The DNA expression ladder of MFGE8 in wild-type and MFGE8-knockout cells (CT26 and MC38) was detected by agarose gel electrophoresis. (B) The mRNA expression of MFGE8 in wild-type and MFGE8-knockout cells (CT26 and MC38) was detected by RT-qPCR. (C) The size and concentration of EVs derived from MFGE8-knockout CT26 and MC38 were measured by using nano-flow cytometry. (D) The small EVs (exosomes) positive markers CD9, CD81, TSG101, and large EVs marker Mitofilin EVs derived from MFGE8-knockout CT26 and MC38 were detected by Western blot. Data was shown in mean ± SEM of n=3 independent experiments per condition. NS = no significant difference, ***P*<0.01, Student’s t-test.
