# Supplementary material for: Extracellular vesicles containing MFGE8 from colorectal cancer facilitate macrophage efferocytosis
Source: Cell Commun Signal. 2024 May 27;22:295. doi: 10.1186/s12964-024-01669-9 (PMC11131254; doi:10.1186/s12964-024-01669-9)

Full unedited blots for Figure 2C

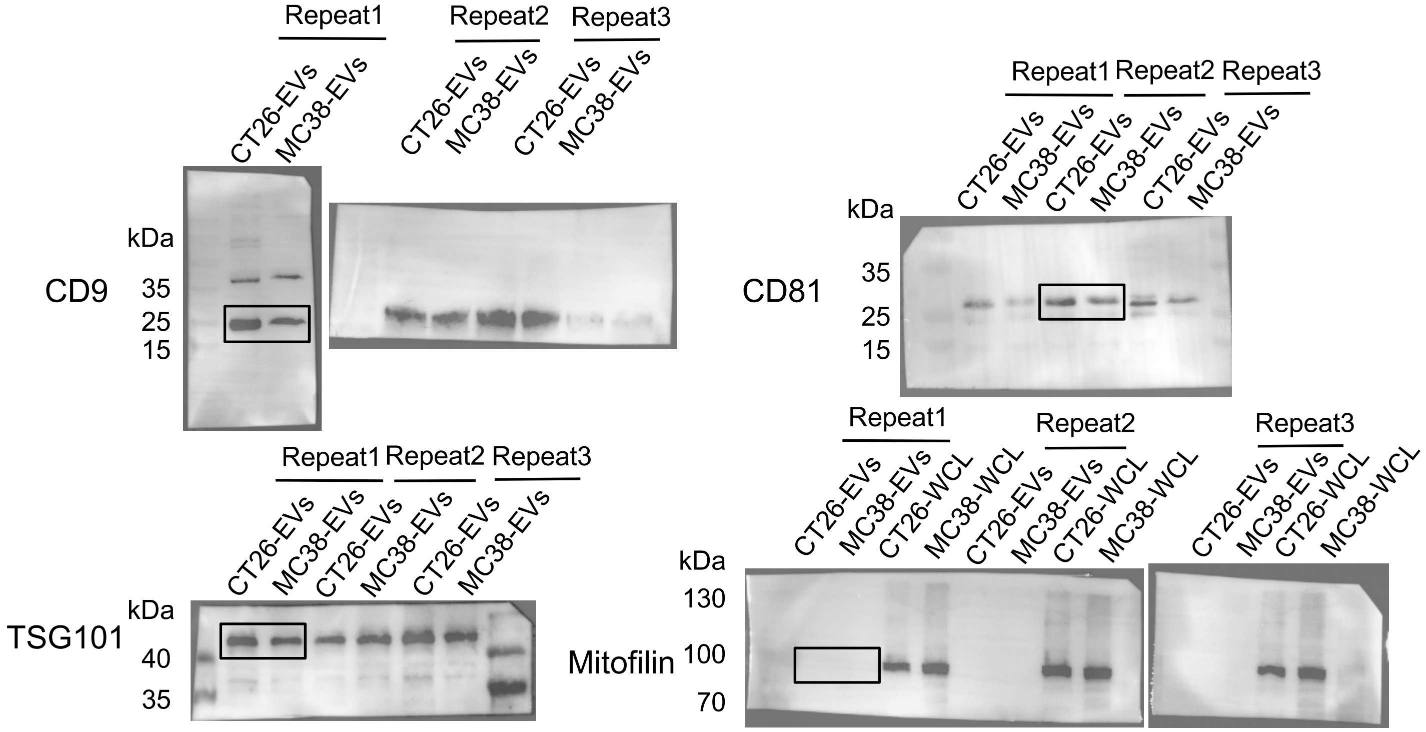

Full unedited blots for Figure 3B

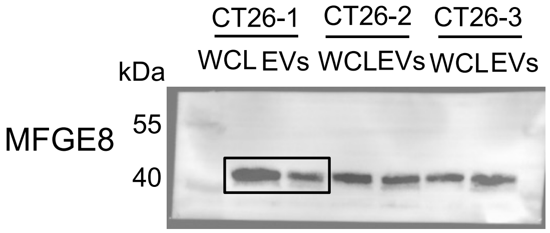

Full unedited blots for Figure 3C

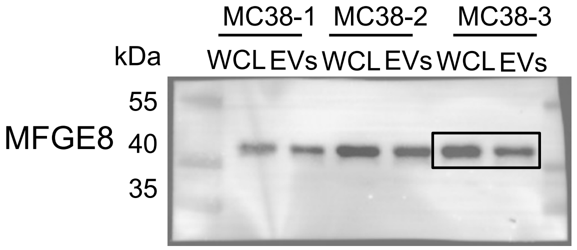

Full unedited blots for Figure 3D

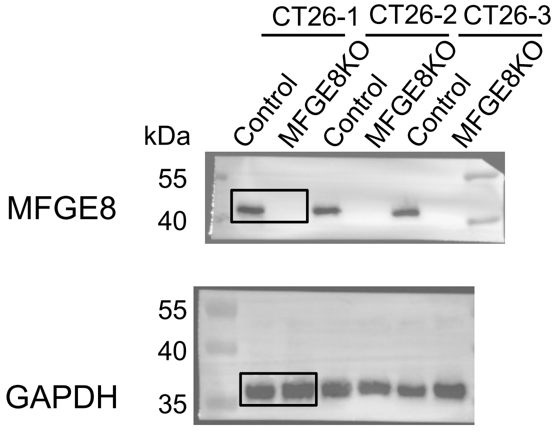

Full unedited blots for Figure 3E

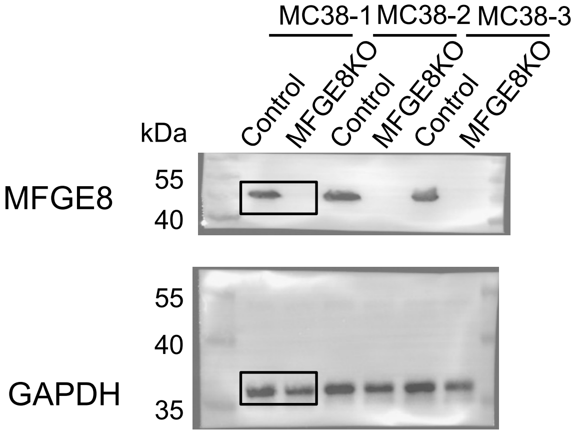

Full unedited blots for Figure 6A

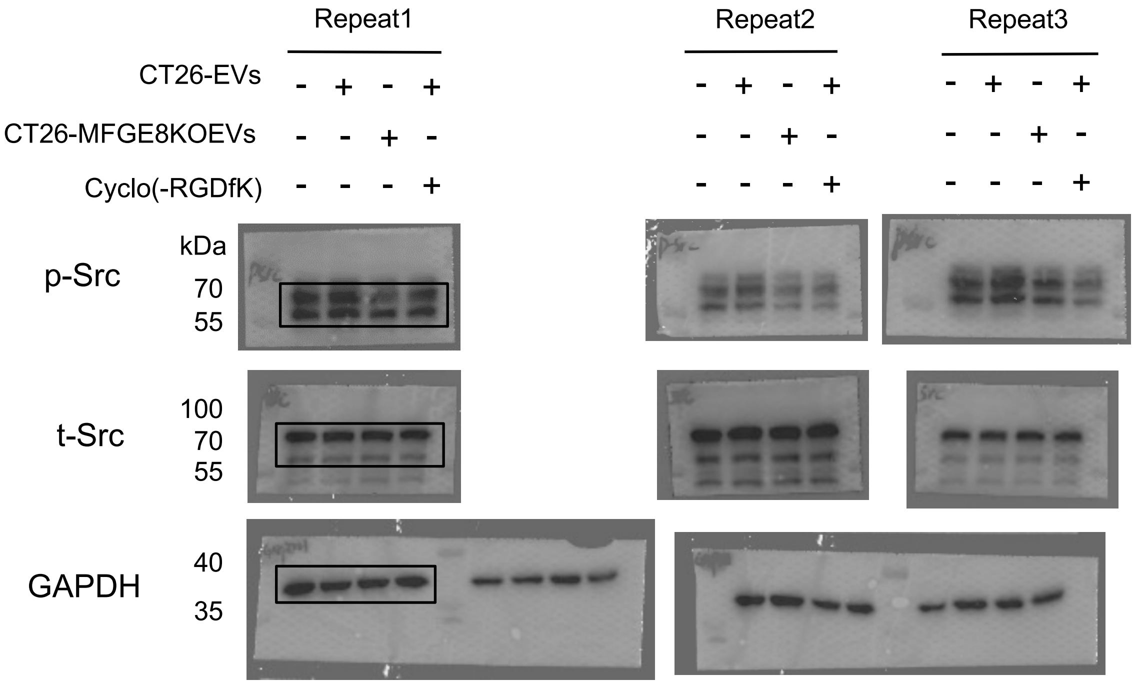

Full unedited blots for Figure 6B

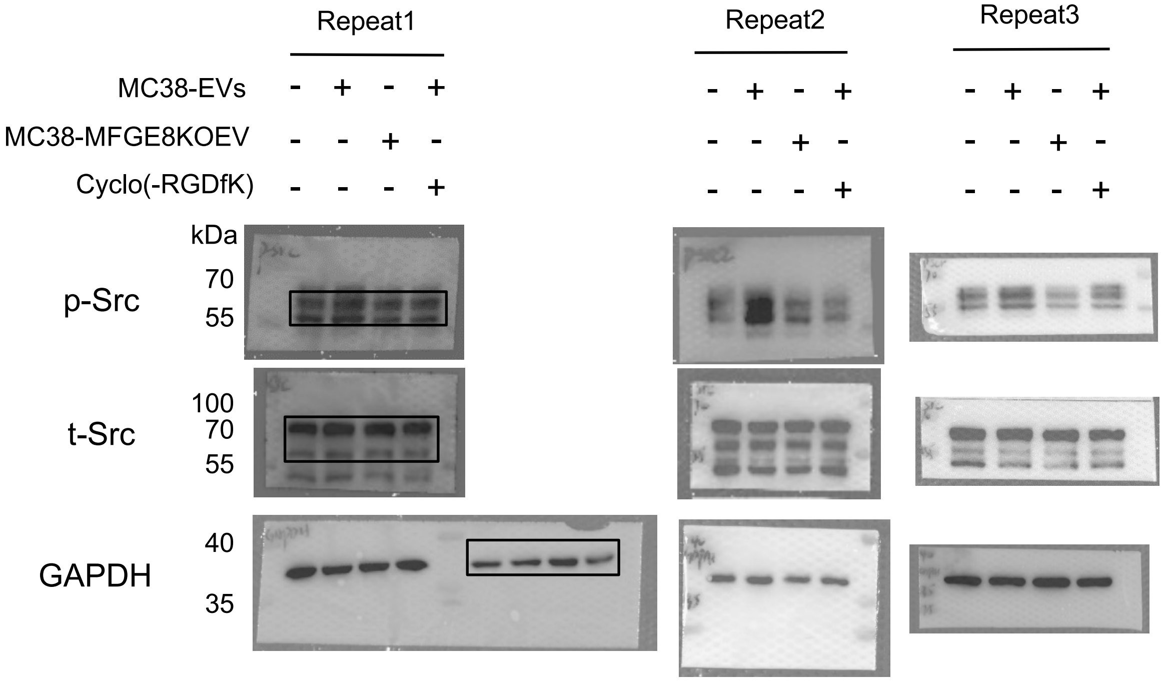

Full unedited blots for Figure 6C

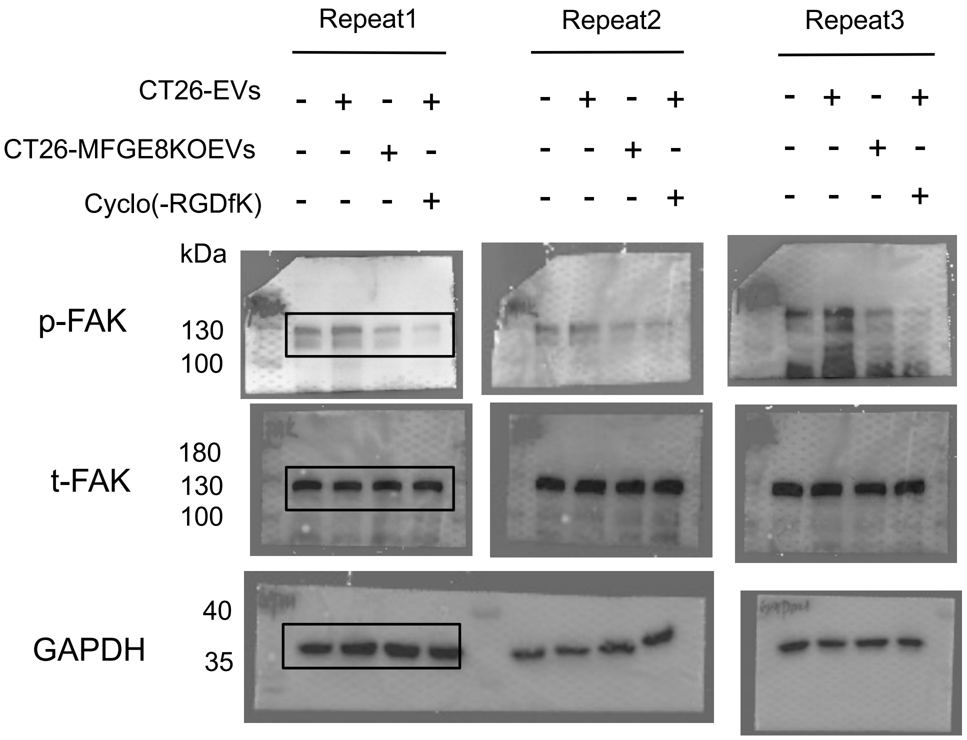

Full unedited blots for Figure 6D

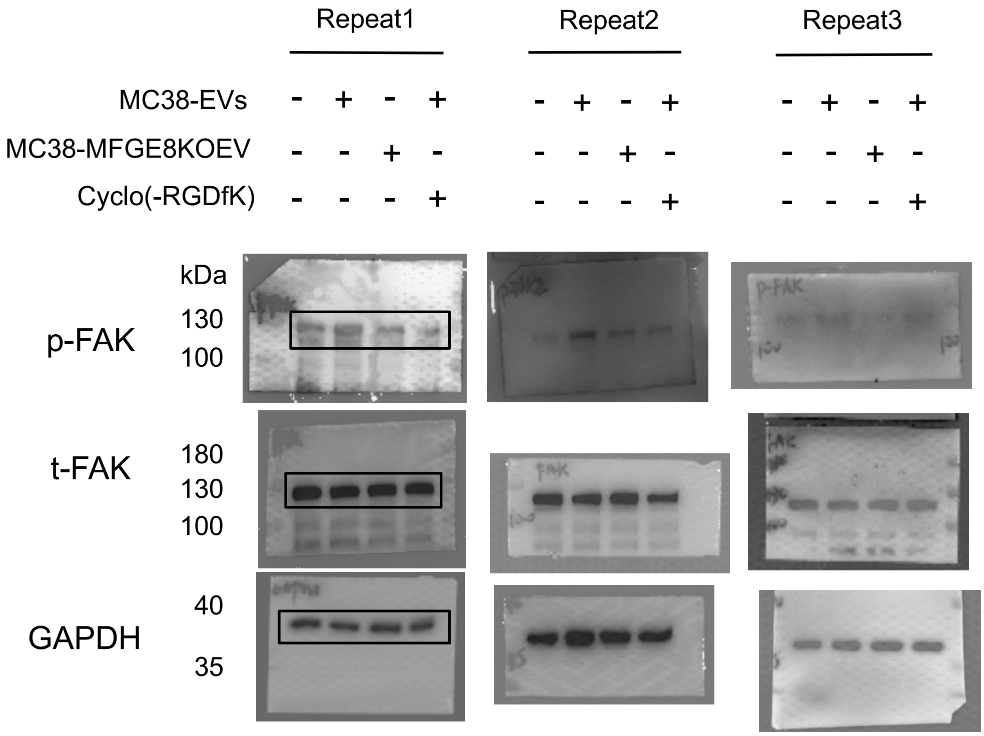

Full unedited blots for Figure 6E

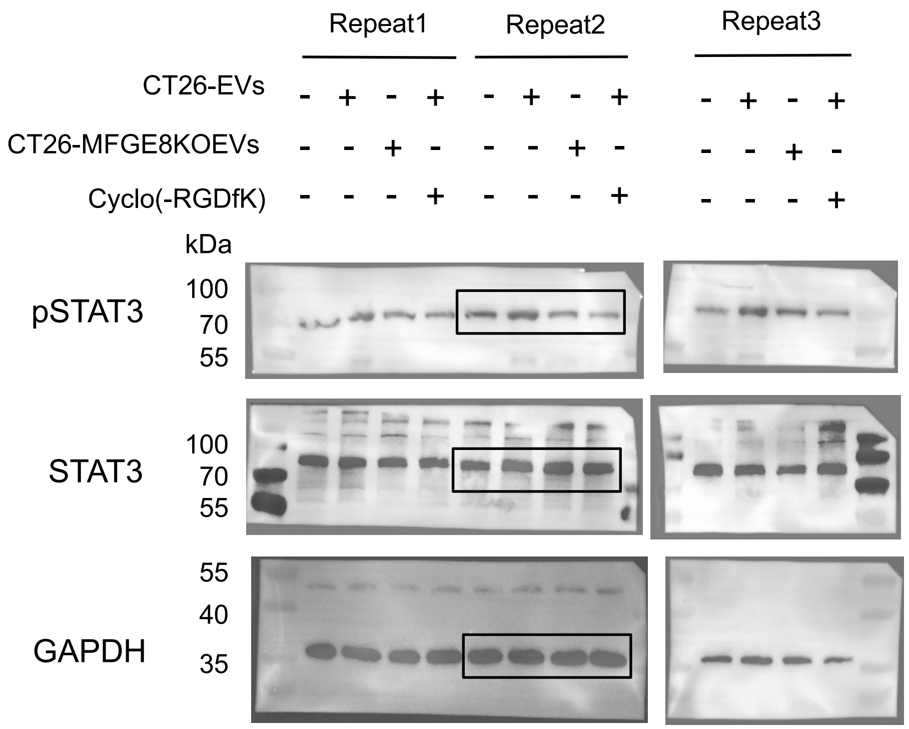

Full unedited blots for Figure 6F

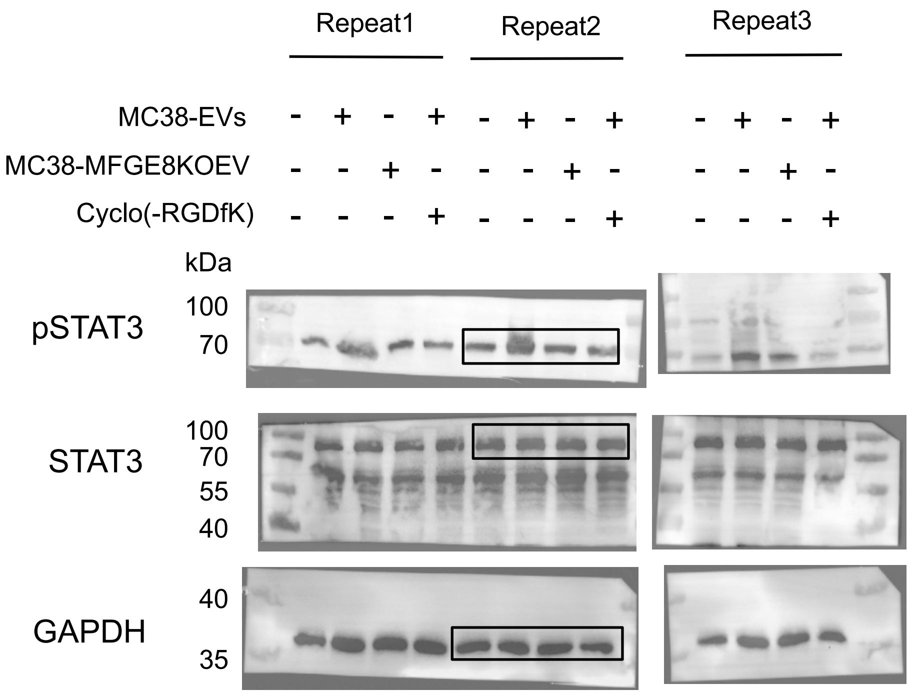

Full unedited blots for Figure S3A

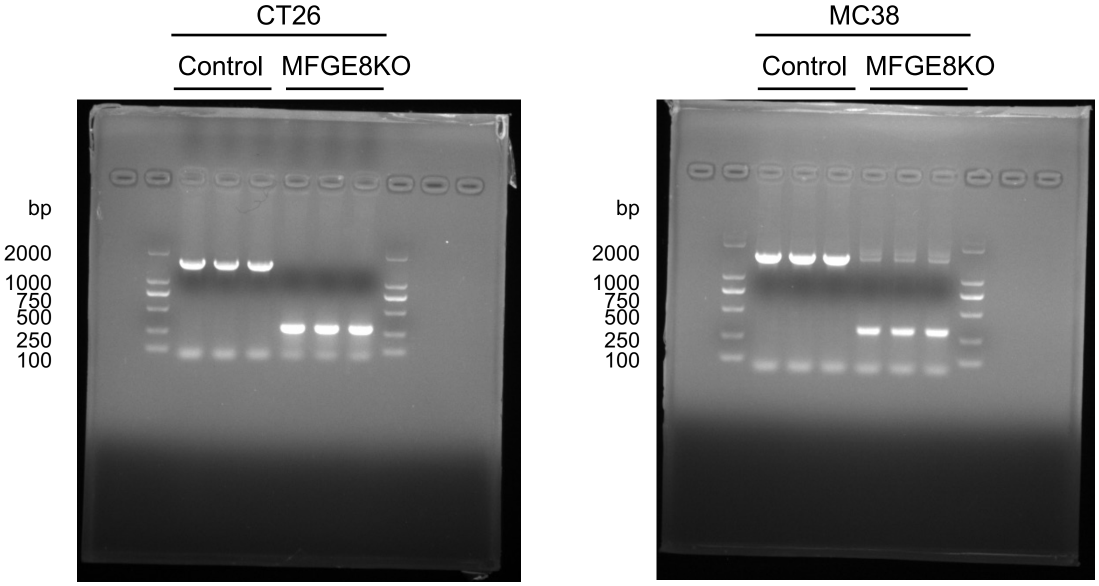

Full unedited blots for Figure S3D

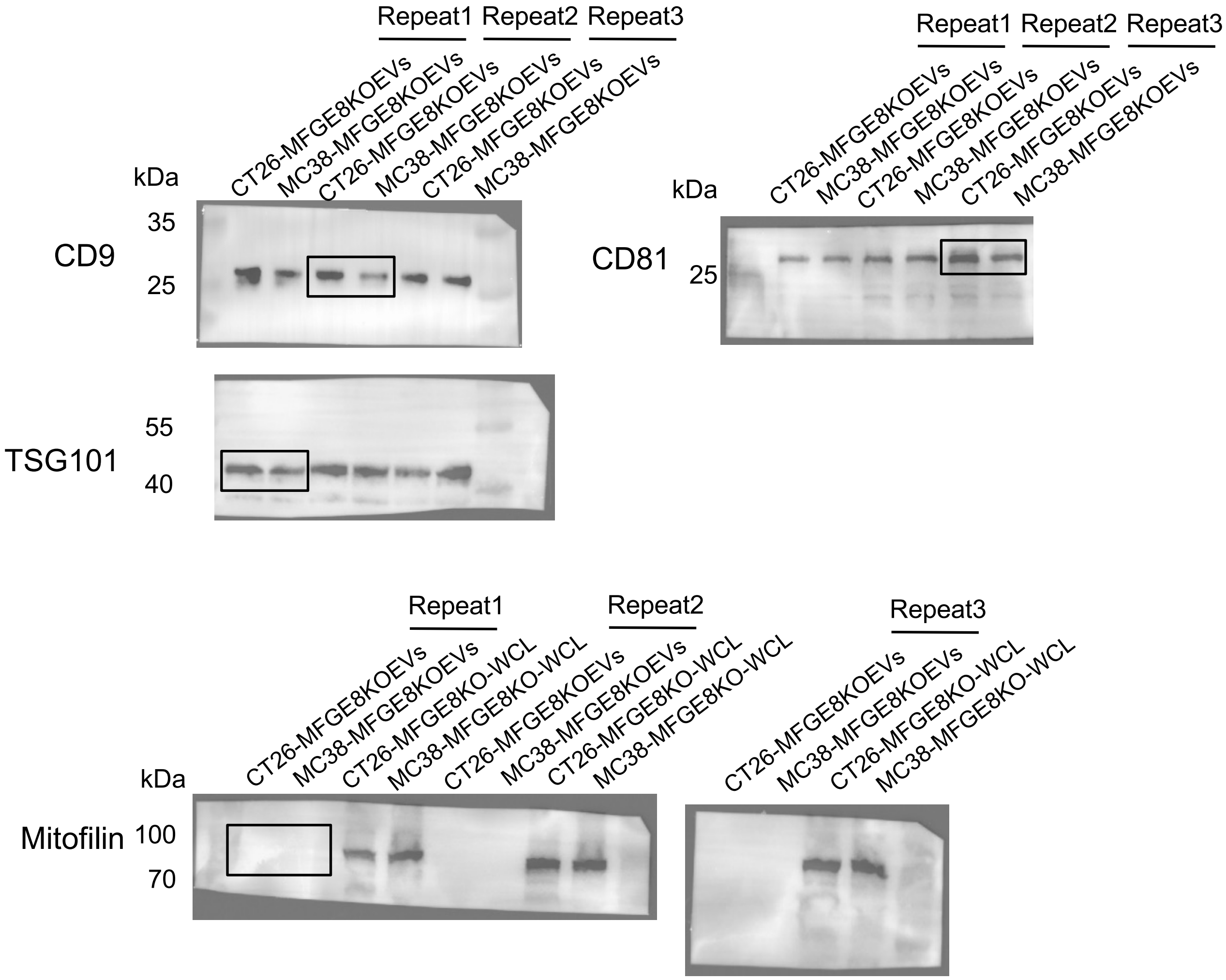

Supplement: Supplementary file 4 — Supplementary Material 4 - Uncropped Western Blot [file 12964_2024_1669_MOESM4_ESM.pdf]
